# Mental health within ESG frameworks: a narrative and descriptive cross-national analysis

**DOI:** 10.3389/fpubh.2026.1741833

**Published:** 2026-03-19

**Authors:** Emanuela Resta, Giancarlo Logroscino, Preethymol Peter, Alberto Costantiello, Angelo Leogrande

**Affiliations:** 1 Dipartimento di Metodi e Modelli per l’Economia, il Territorio e la Finanza, Università degli Studi di Roma La Sapienza, Rome, Italy; 2 Dipartimento di Ricerca Clinica in Neurologia, Università degli Studi di Bari Aldo Moro, Bari, Italy; 3 Dipartimento di Ingegneria dell’Innovazione, Università del Salento, Lecce, Italy; 4 Dipartimento di Management, Finanza e Tecnologia, Università LUM Giuseppe Degennaro, Casamassima, Italy; 5 Dipartimento di Management, Finanza e Tecnologia, Università Lum Jean Monnet, Casamassima, Italy

**Keywords:** descriptive analysis, ESG dimensions, governance quality, mental health, policy-oriented review, sustainability

## Abstract

Despite the growing use of environmental, social, and governance criteria for evaluating sustainability and institutional performance, mental health remains at the periphery of mainstream discourse on the topic. The existing literature often explores environmental efficiency, social inclusion, and good governance without necessarily incorporating mental health as a core component of the sustainability framework. This research study helps fill this existing research gap by integrating a narrative literature review with a cross-country descriptive analysis of the associations between the various dimensions of ESG factors and mental health outcomes across 31 countries from 2010 to 2022. Mental health outcomes are measured by population-level prevalence rates reported by the World Health Organization, while the various ESG factors are extracted from the World Bank’s Sovereign ESG dataset. This research study will employ a thematic-descriptive research design to examine the associations among the multiple aspects of the ESG framework and mental health outcomes, without necessarily attempting to build a theory or construct a model of causation. This study will not attempt to apply the research results to create a specific behavioral or welfare-theoretic framework or model of mental health outcomes. Instead, this research study will aim to provide a broad overview of the associations between the various factors of the ESG framework and mental health outcomes across 31 countries. Among indicators of good governance, the rule of law consistently plays an essential role in shaping mental health outcomes across the analyzed countries.

## 1 Introduction

Mental health is a core but complex concern in current public health discourse that has long remained fractured across inter/ transdisciplinary lines. Although extensive research has identified the determinants of psychological wellness, the concern of mental health has too often been studied in relation to sustainability considerations in the broad sense independently. At the same time, the environmental, social, and governance (ESG) framework has come to be widely used to evaluate sustainability performance and institutional quality in countries, corporations, and public bodies. Notwithstanding its current salience, research on mental health has remained marginally represented in studies following the ESG framework in which it is implicitly subsumed by generic considerations of the social type rather than being treated as a distinct policy outcome in its own right. In light of these considerations, the current research seeks to address the current gap in the literature by systemically compiling and integrating evidence on the interconnection between mental health and the various dimensions of ESG considerations. Rather than proposing an explicit welfare or theoretical framework in its own right, the research framework of the current investigation therefore adopts an integrative narrative approach to consider how the various dimensions of mental health are empirically connected to considerations of environment, society, and governance in various countries. In so doing, it is proposed that the current research can provide further insight on how different bodies of research that have long remained isolated in distinct silos of research can be understood in relation to each other in the framework of sustainability considerations in particular. Research has identified particular interconnections between various dimensions of mental health and individual considerations of the ESG type in particular but independently of each other. Research in the area of environment has identified interconnections between various dimensions of pollution and climate change on the one hand and dimensions of mental health on the other in particular without being situated in the framework of sustainability considerations in particular (1). Research in the area of social considerations has identified interconnections between dimensions of inequality, work conditions, housing insecurity, and dimensions of social exclusion on the one hand and dimensions of mental health on the other in particular without being situated in the framework of sustainability considerations in particular (2). Research in the area of governance has identified interconnections between various dimensions of healthcare regulation, welfare considerations, legality considerations, and digital governance on the one hand and dimensions of mental health on the other in particular without being situated in the framework of sustainability considerations in particular (3).

### 1.1 Welfare framework: mental health, ESG, and social well-being

In the context of the current research, mental health is explored in the context of a welfare approach which is both descriptive and interpretive in nature. This approach considers mental health as an essential part of overall welfare and sustainable development, consistent with findings that link environmental governance and social welfare conditions to improved well-being at the population level (4). This approach is not a welfare and optimization theory per se but is used as an organizing principle for the interpretation of existing knowledge on the relationship between mental health outcomes and overall socio-economic factors. As opposed to the approach taken in studies which only consider mental health issues in the context of clinical and epidemiological models of health and illness, the current research takes a socio-economic approach which focuses on the capacity of society to provide an enabling environment for mental health. With this descriptive focus in mind, the environmental, social, and governance (ESG) factors are seen as descriptive contexts that define the characteristics of conditions related to sustainability, as opposed to being tools of the welfare outcome process itself. From this perspective, the environmental factor is defined as descriptive conditions of observed vulnerability to environmental stressors, the availability of repairative environments, and the nature of sustainability transitions that might affect psychological well-being (4). This descriptive focus encompasses the social factor as well, defined as descriptive conditions of observed inequalities, working conditions, housing security, the availability of resources, and the opportunities of the life course that have been traditionally linked to the outcome of psychological well-being in the empirical literature (5). These conditions are viewed as cumulative, but also as mutually related, in that they have been observed to occur in combination, but in varying degrees of psychological well-being, across the life course. Governance is viewed as the facilitating, moderating context that conditions the translation of environmental and social factors into psychological well-being. Within the narrative focus, the descriptive conditions of the quality of governance, as defined by the effectiveness of institutions, the strength of regulation, and the supply of public goods, are viewed as conditions that affect the stability, predictability, and inclusiveness of the process of sustainability (6). Within this narrative focus, the outcome of psychological well-being is defined as the cumulative effect of the functioning of environmental sustainability, the process of social inclusion, and the conditions of good governance, as opposed to the outcome of a distinct process of welfare maximization (5, 6).

### 1.2 Descriptive pathways linking ESG conditions and mental health

ESG Indicators from a Descriptive Perspective on Sustainability and Mental Health. In the current study, the role of the ESG framework is that of a descriptive tool for a set of sustainability indicators, as opposed to an economic entity or a depiction of the decision-making processes at play within the context of sustainability economics. The application of the ESG indicators serves as a means for summarizing the descriptive conditions within various countries, as a way to systematically describe the sustainability conditions that have been linked to the descriptive mental health performance (7). The indicators from the Sovereign ESG Database, developed by the World Bank, act as a descriptive means for the systematic comparison across various countries (8). In the context of the descriptive analysis, the indicators serve as a means for the systematic interpretation related to the descriptive conditions within the context of sustainability, as opposed to the economic choices, limitations, or optimization related to the economic optimization process (7). The application of the ESG indicators within the cross-country context allows for the identification of the association patterns related to the mental health performance within the context of the various countries (9). The application of the econometric analysis within the context of the study serves as a means for the systematic analysis related to the descriptive conditions, as opposed to the economic optimization process or the economic choices within the context of sustainability economics (7). In the context of the descriptive analysis, the current study allows for the assessment related to the sustainability conditions as they pertain to the descriptive mental health performance (7–9).

### 1.3 ESG indicators in a descriptive sustainability–mental health perspective

In the current analysis, the role of the ESG framework is that of a descriptive tool for a set of indicators of sustainability, as opposed to an economic entity or a proxy for decision-making processes. The use of the ESG factors serves as a tool to summarize the observable conditions across countries, making possible the structured representation of the sustainability environments related to the observable mental health outcomes (7). The indicators extracted from the Sovereign ESG Database, created by the World Bank, represent a standardized tool for the purpose of the descriptive analysis of the comparative evaluation across countries (8). The indicators used within the analysis are considered to reflect the observable conditions of the sustainability factors related to the energy systems, the environment, the social systems, or the governance systems, as opposed to economic choices or optimization decisions. By incorporating the use of the ESG indicators within the framework of the comparative evaluation across countries, the current analysis finds the correlation between the use of the ESG factors and the population mental health performance outcomes across the nations (9). The use of the econometric analysis serves as the methodological tool within the analysis with the purpose of evaluating the observable patterns within the structured framework of the analysis, as opposed to the evaluation of the economic decision-making or optimization process within the framework of the construct of the sustainability economics. From this standpoint, the current analysis serves as the tool for the evaluation of the sustainability environments within the framework of the changes within the observable mental health performance across the countries (7–9).

### 1.4 Descriptive and integrative contributions to the ESG–mental health literature

A growing body of literature has investigated the link between ESG factors and mental health, tending to focus on individual elements in isolation (10). Nevertheless, the existing literature reviews, as well as the conceptual debates, have remained broadly disjointed, corresponding to the boundaries between different subject fields, with no integrated approaches (11). It is with this background that the current work contributes to the existing literature in multiple descriptive and interpretative ways. Firstly, the article sets forth an integrated narrative account of the existing findings on the link between ESG factors and mental health, integrating the environmental, social, and governance approaches into an encompassing descriptive framework (10). Rather than introducing an innovative analytical framework, the narrative account attempts to synthesize the findings into an encompassing framework, which have been analyzed individually across the different threads of the existing literature. Secondly, while the existing literature is broadly based on the narrative summaries, the current work supplements the narrative account with new cross-country empirical evidence, allowing the systematic examination of the described ESG factors and the mental health findings. This empirical part is intended to assist the interpretative process, rather than providing an innovative framework (12). Thirdly, the article sets forth an interpretative account of mental health, which is not only an outcome measured across the population, contingent on the described ESG factors, but also an indicator, which is relevant to the sustainability debates (11). This account does not advance an innovative sustainability hypothesis, but rather focuses on the meaningful incorporation of mental health into the ESG-oriented framework (10). Fourthly, the empirical part highlights the importance of institutional factors, specifically the rule of law, which have been broadly recurring elements across the described link between the ESG factors and mental health. While the importance of the institutional factors, specifically the rule of law, have been broadly discussed in the different literatures, their incorporation into the narrative account, specifically into the encompassing framework, which describes the link between the ESG factors and mental health, have remained broadly under-explored (11, 12).

### 1.5 From optimization to context: a narrative framework linking ESG and mental health

However, as a research approach, a specific choice is made within this research not to identify a representative economic actor or formulate an explicit objective function. Mental health is neither treated as a result of individual or collective optimization nor as a result of a rational choice process subject to constraints as implied by conventional welfare or utility maximization models of economy (13). Mental health is treated as a macro-indicator that arises from the combination of environmental factors and structural governance processes rather than as a result of processes deeply rooted within micro-economic decision processes that are subject to constraints as implied by conventional models of economy (10). In this framework, countries are not represented as optimizing agents, nor are ESG factors represented as policy tools to optimize welfare. Rather, factors of environment, social factors, and governance are used to describe the contexts in which the prevalence of mental health is noted. In this line of thinking, the prevalence of mental health can be described as the outcome of underlying factors of psychosocial stress and the capacity of health systems to identify and report cases of mental health (14). Therefore, differences in health outcomes are noted in relation to differences in structural factors rather than behavioral factors that are responsive to incentives (10, 13). Through the use of a descriptive and storytelling method, it is hoped that this research will be able to discover some systematic patterns and typologies which reveal the co-movements between ESG variables and outcomes related to mental health, without having to rely heavily on causality and optimization. The focus of this analysis is therefore placed on interpretation rather than welfare maximization, which will allow mental health to become a constituent element of sustainability analysis rather than a byproduct or aftermath (14).

### 1.6 A descriptive and exploratory analysis of ESG indicators and mental health across countries

This paper uses a descriptive and exploratory empirical approach in an attempt to describe the patterns of associations between mental health prevalence and environmental, social, and governance (ESG) factors. Rather than attempting to estimate causal effects or test hypotheses based on behavioral or welfare theories, this study focuses more on describing patterns of associations, co-variations, and cross-country differences based on the available macro-data (15). Mental health prevalence in this study is thus treated as an aggregate outcome reflecting broader environmental, social, and governance conditions, but it is not modeled as an outcome of economic optimization by an economic agent, nor as an outcome of a specific causal process (16). Rather, it can be viewed as an aggregate outcome reflecting broader conditions. The approach used in this study combines panel regressions with clustering analysis in an attempt to describe the multidimensional patterns. Panel regressions are used in an attempt to describe patterns of associations between mental health prevalence and some ESG factors, while clustering analysis groups countries into clusters based on structural patterns along sustainability factors (17). Taken together, this approach enables the mapping of patterns without relying heavily on theoretical assumptions. More specifically, this study does not use any of the standard approaches in economics for isolating exogenous variation in the data, such as instrumental variables, natural experiments, or policy discontinuities. As such, the study does not estimate causal effects between ESG factors and mental health prevalence, but instead focuses more on a narrative interpretation of the patterns in line with the broader approach of synthesizing evidence across environmental, social, and governance factors (15, 17). Adopting this approach enables this study to contribute to the literature by attempting an empirical description of patterns of associations between mental health prevalence and factors associated with sustainability in a way that explicitly acknowledges the limits of correlational analysis (16).

### 1.7 Analytical scope and interpretive strategy

Because of the intrinsically multi-dimensional nature of the environmental, social, and governance (ESG) issues at the center of this research, the approach taken in this work privileges internal consistency in the research questions and methods of inquiry and interpretation over the construction of a comprehensive theory and the use of formal modeling (18). This is because the phenomena studied in relation to the literature on ESG issues cover a wide spectrum of social and institutional processes in the environment and society at which the mental health of individuals is affected in various ways and over which time scales that stretch beyond the immediate term of the data (19). In such a context, the imposition of a comprehensive theory and welfare models may lead to the results being overstated in relation to the data and may lead to the imposition of an unnecessary level of complexity in the underlying theories and models of the data. Therefore, the approach in this work focuses on the identification and interpretation of the patterns of association and cross-country variation in the data without the imposition of a comprehensive theory and welfare models. This approach maintains a solid foundation in the underlying theory and models of the data and focuses on the underlying institutions and incentives of the mental health of individuals in relation to the various aspects of the ESG issues without overstating the results in relation to the data and the underlying models of the data (18–20).

### 1.8 Originality and contribution of the study

Originality in this paper primarily lies in the integration and interpretation of existing evidence, rather than in the application of a new theoretical approach or causal identification design. While a significant body of literature has explored links between environmental factors, social inequality, governance quality, and mental health, these findings are usually scattered across fields of study and explored in separate research. This paper contributes to this body of knowledge by offering an integrated interpretation of these threads through the prism of the ESG approach, which is explicitly viewed in this paper as a descriptive, contextual device rather than a causal model. Another aspect of originality comes from the combination of a narrative, interdisciplinary literature review with an exploratory cross-country quantitative study. While the quantitative study in this paper does not seek to establish causal links but instead seeks to identify patterns of recurrence which are supportive of pre-existing themes in the literature, it can be argued that this study contributes to this body of knowledge by adding an extra layer of meaning to the narrative literature review, thus improving the internal consistency of this paper. Finally, this paper contributes conceptually by treating mental health as a cross-cutting contextual factor for sustainability conditions, rather than as an outcome within a specific sector. While this approach widens the scope of interpretation for research related to ESG factors, it does so in a cautious, disciplined way which corresponds with the descriptive nature of the evidence used in this paper.

## 2 Literature review

However, the incorporation of mental health issues explicitly within conventional frameworks of environmental, social, and governance (ESG) analysis is currently patchy and fragmented even within the growing body of research on mental health within public health studies and the wider sustainability debate (15). In conventional ESG analysis, environmental, social, and governance factors are treated as distinct domains of analysis, within which mental health factors are merely incidentally or subsidiarily mentioned as a consideration rather than a defining aspect of sustainability (2). The literature review undertaken within this research is a narrative review with a descriptive approach that seeks to integrate mental health as a consideration across the three domains of ESG analysis without necessarily developing a conceptual framework or causal hypotheses about mental health within the existing body of literature. In the environmental dimension, previous studies have identified a series of links between outcomes of mental health and exposure to risk and protective factors in the environment. Distress, as a negative aspect of psychological well-being, has been repeatedly associated with exposure to pollution, climate change, or degraded environments, while positive aspects of emotional regulation, resilience, or well-being have been associated with access to green or blue spaces or nature-based environments (1). Significantly, this body of knowledge stresses that these benefits are non-uniform across different age groups, health status, mobility, or accessibility. With respect to the social component of ESG, mental health is more overtly related to lived experience. The literature emphasizes the cumulative and persistent nature of psychological problems related to housing insecurity, food insecurity, discrimination, migration uncertainty, precarious work, and a lack of social support (2). These works tend to place mental health within a structural inequality and social stress continuum, as opposed to identifying isolating factors. Governance research highlights the institutional contexts in which environmental and social risks are addressed. The quality of governance is conceived as a contextual factor influencing susceptibility to risk, insecurity, and protection, but not as a cause of mental health outcomes. Differentials in mental health prevalence are presented in relation to contexts varying in regulatory strength, transparency, and effectiveness. Outcomes are not traced back to policy tools. In order to weave these threads together, this paper uses a narrative synthesis with a focus on the interdisciplinary literature from the fields of public health, environment, the social sciences, and public policy. Articles included in the narrative synthesis included those relevant to mental health issues as they relate to at least one dimension of ESG. This narrative synthesis is interpretive, aiming to place mental health issues into the broader narrative of ESG discussions, without making any claims to determining the bias.

### 2.1 Mental health within the environmental dimension of ESG: a narrative review of urban and nature-based contexts

A narrative review of the Environmental (E) dimension of ESG factors was carried out to synthesize the conceptualization of mental health outcome studies that have previously been conducted in relation to green conditions. This narrative review provides an explanatory summary of the salient themes, relationships, and trends that have emerged in recent literature on the association between sustainability and mental health. The main source of the literature reviewed in this text is peer-reviewed publications indexed in Scopus, which is preferred for its ability to cover a wide range of interdisciplinary studies in the areas of public health, environmental sciences, urban planning, and health policy. The literature reviewed is mainly based on contributions that contain a discussion of mental health outcomes in relation to environmental exposure, green infrastructure, nature-based solutions, and environmental sustainability. One of the most prominent themes that emerge in the reviewed literature is the role of natural and green environments in the maintenance of mental well-being. Many studies report the relationship of access to urban green spaces, walkable green corridors, biodiverse soundscapes, and the reduction of stress, depression, and psychological distress, and the prevention of severe mental health outcomes (21–24). However, it is also important to note that the relationship is not the same for all individuals, and it varies in terms of age, health, and social vulnerability, indicating that the relationship is environment-dependent (25, 26). Some research also highlights the significance of the interaction between the individual and the environment. Mental well-being is defined as being affected not only by the presence of nature but also by the use of nature through walking or other means of active mobility (27, 28). Another strand of the literature focuses on the process of environmental sustainability. Studies on the process of eco-digital transformation, energy system change, and the governance of green infrastructure portray the process of sustainability transitions as having the potential for positive impacts on mental health in the long term while, at the same time, creating short-term stress (29, 30). This strand of the literature informs an interpretative understanding of the process of environmental sustainability. The narrative review also points to an emerging literature on environment-related and nature-related interventions. Research on horticultural therapy, nature-related activities, and multisensory stimulation by natural elements has been linked with decreased symptoms of depression, anxiety, and stress, conceptualized as an enabling and preventive resource for mental health (31, 32). Research related to this domain in Environmental Psychology and Biomedicine delves into further potential biologically and physiologically linked concepts of stress and related findings emerging from the Microbiota-Gut-Brain domain (33, 34). On a more general level, there is a clear consensus among the reviewed literature on a narrative description of mental health as a result of the interplay between environmental quality and patterns of exposure, as well as wider sustainability transitions. In other words, rather than isolating causal factors, these studies can all be seen as contextualizing mental health within environmental systems as a whole, as part of the Environmental pillar within the ESG framework. This narrative approach is incorporated into the empirical analysis that is presented below. See Table 1.

**Table 1:** Narrative synthesis of environmental dimensions linking ESG and mental health.

Narrative theme	Scope of the literature	Illustrative references (Year)	Predominant study types	Recurring descriptive patterns	Interpretive relevance for mental health
Green infrastructure and urban contexts	Urban green spaces, planning strategies, spatial equity, scenario-based sustainability	Israelsson et al. (29); Pastore et al. (126); Lahoz et al. (127); Gao et al. (25); Li et al. (128)	Cross-sectional population studies; spatial analyses; planning-oriented case studies	Studies consistently describe associations between access to green infrastructure and lower levels of psychological distress across urban contexts	Urban green environments are repeatedly discussed as supportive contexts for mental well-being, particularly when access is equitably distributed
Daily exposure, mobility, and environmental experience	Walking environments, daily mobility patterns, sequential exposure, soundscapes	Tu et al. (22); Ma and Kwan (28); Cheng et al. (129); Egerer et al. (130); Chen et al. (27)	Mobility tracking studies; exposure-based observational designs	The literature emphasizes that mental health outcomes are shaped not only by the presence of green spaces but also by how individuals encounter them in daily life	Mental well-being is framed as sensitive to lived environmental experience, with active mobility and multisensory exposure frequently highlighted
Nature-based interventions and vulnerable groups	Horticultural therapy, ageing populations, students, children, post-crisis resilience	Prabhu et al. (131); Xu T. et al. (2026); Tate et al. (31); Suhendy et al. (132); Yang et al. (133); Muhammad et al. (134); Fry et al. (135)	Intervention studies; cohort studies; mixed-method designs	Across diverse contexts, studies describe beneficial associations between nature-based interventions and reduced distress among vulnerable groups	Nature-based interventions are narratively positioned as supportive and preventive resources for mental health in high-risk or sensitive populations
Environmental transitions, technology, and biological interfaces	Sustainable transitions, eco-digital systems, biomonitoring, microbiota–gut–brain research	Zhang et al. (30); MEDES (158); IDEAL, (159, 160); Martinez-Moral and Kannan (33); Pan et al. (34)	Experimental and exploratory studies; technological and biological assessments	The literature portrays sustainability transitions as complex processes associated with both adaptive benefits and transitional stress	Mental health is discussed as intertwined with technological, biological, and systemic change, highlighting dynamic and non-linear relationships

This table summarizes recurring themes in the literature through a descriptive narrative synthesis. It organizes empirical findings by thematic focus and interpretive relevance, without assessing causal effects, ranking evidence strength, or proposing formal analytical frameworks.

### 2.2 Social dimensions of ESG and mental health: a narrative review

The literature review on the Social (S) aspect of ESG uses a narrative-descriptive approach with the objective of synthesizing the manner by which mental health outcomes have been related to social structures, conditions, and institutions by previous studies. Rather than striving for exhaustiveness or hypothesis-testing, the review focuses on theme-identification, interpretation frameworks, and patterns by which mental health has appeared as a social phenomenon. The literature review is based on peer-reviewed studies that are part of the Scopus database with a specific focus on contemporary studies that are currently related to debates on social sustainability as a mental health issue. The literature review includes a wide range of studies on the Social Sciences that approach mental health as a phenomenon affected by social infrastructure, inequalities, and conditions rather than as a purely individual issue. In all studies reviewed, mental health is consistently treated as a phenomenon situated within social arrangements and infrastructure related to emotions. Studies on migration indicate that uncertainty related to legality, economy, and existence is associated with greater psychological distress (35). Studies on water insecurity indicate that insecurity is simultaneously a source of social conflict as well as a cause of mental strain (36). Social isolation and social loss are common factors that are treated as sources of disadvantage related to biological acceleration as well as a decrease in psychological well-being (37). A common theme among the studies is related to inequalities on social as well as economic infrastructure. Studies on longitudinal cohorts indicate that prolonged disadvantage related to economic segregation, food insecurity, as well as housing instability is associated with disproportionate mental health outcomes (38–40). These sources are typically related to gender, class, as well as a lifecycle perspective that produces disproportionate mental health-related depression, cognitive impairments, as well as psychological distress (41). In a parallel manner, there is documentation on the role of social buffers that protect against mental health-related distress. See Table 2-

**Table 2:** Narrative synthesis of social dimensions linking ESG and mental health.

Narrative theme	Scope of the literature	Illustrative references (year)	Predominant study types	Recurring descriptive patterns in the literature	Interpretive relevance for mental health
Social infrastructures, relationships, and social support	Family relations, community participation, social contacts, leisure, informal support networks	Duff (136); Wasserman and Lögdberg (137); Freeman et al. (138); Duijsens et al. (139); Zou et al. (140); Chang et al. (141); Mason et al. (142)	Longitudinal cohort studies; social network analyses; qualitative and mixed-method designs	Studies repeatedly describe social support, participation, and relational continuity as contexts associated with better psychological well-being across the life course	Social infrastructures are narratively framed as protective contexts that support emotional regulation, cognitive health, and resilience over time
Social inequalities, economic insecurity, and life-course disadvantage	Housing and food insecurity, economic segregation, class and gender inequalities, ageing	Zhou and Lu (40); Cui et al. (38); McGowan (143); Richards et al. (144); Li et al. (128); Patsouras et al. (145); Koomson et al. (146)	Population-based observational studies; life-course and inequality-focused analyses	The literature emphasizes cumulative exposure to disadvantage and stress as recurring features shaping uneven mental health trajectories	Mental health is interpreted as socially patterned, reflecting long-term exposure to inequality and economic insecurity rather than isolated individual factors
Marginalization, stigma, and institutional exclusion	Migration, homelessness, minority stress, healthcare access, carceral and institutional systems	Rast et al. (35); Malenfant (147); Ameen et al. (148); Barbee & McKay (41); Nezamdoust & Ruel (149); Carter et al. (47); Gerber et al. (150)	Qualitative studies; institutional and policy analyses; cohort and case-based designs	Across contexts, studies describe how stigma and exclusion embedded in institutions shape experiences of distress and vulnerability	Mental health outcomes are discussed as contingent on institutional environments that regulate access, recognition, and protection
Crisis, stress exposure, and psychosocial resilience	Pandemic effects, social disruption, uncertainty, biological stress responses	Mir Mohamad Tabar et al. (36); Kwon & Kim (37); Stoffel et al. (151); Soares et al. (39); Moreno-Agostino et al. (152); Feng et al. (153); Mahran et al. (154); Muyingo et al. (155)	Longitudinal crisis studies; stress-related biomarker research; population surveys	The literature portrays mental health during crises as shaped by interactions between stress exposure, social position, and adaptive resources	Resilience is interpreted as a socially mediated process, dependent on protection systems, equity, and collective capacity to absorb shocks

This table presents a narrative and descriptive synthesis of the literature on the social dimension of ESG and mental health. It organizes recurring themes and interpretive patterns without ranking evidence, assessing causal strength, or proposing formal analytical models.

### 2.3 Narrative search strategy and study selection for governance and mental health

The literature that deals with the Governance (G) factor of ESG is examined in a narrative and descriptive manner that aims at synthesizing how mental health outcomes are considered within a governance discourse. Instead of building and testing models of governance or specifying mechanisms of causation, this review aims at recording how mental health is considered as an outcome situated within institutional settings and mechanisms of governance. The narrative review aggregates the results of peer-reviewed studies indexed on the Scopus database through targeted searches conducted with the keywords mental health AND governance. In order to reflect the most current discourse on the topic, the review focuses on articles published within the year 2026. This search generates a relatively small pool of 12 studies because of the specialized nature of research at the intersection of the disciplines of governance and mental health. All identified studies are reviewed comprehensively as part of the qualitative analysis because together, they offer a contextual view of the linkage between governance processes and mental health outcomes. In the literature studies examined in this work, mental health is presented as a concern of the healthcare system in general and as an outcome of governance structures in particular. Governance is defined as the contextual mediator between the impact of social-technology-environmental stresses and the level of the population. It is stated that mental health outcomes cannot be ascribed to the institution in question. A strong current of research focuses on governance in connection with digital and technological systems. Zhang et al. (30), for instance, argue that eco-digital governance in China has improved socio-energetic resilience by diminishing systemic uncertainty in times of structural transition and thereby indirectly promoting psychological well-being. Conversely, some research points to the anxiety and mistrust that can be created by the lack of digital governance. Moseley (42) and Hull (43), for instance, critically discuss the use of artificial intelligence in employer-led mental health support and observe that the lack of governance has led to the blurring of care and surveillance boundaries that cause psychological distress. Elyamany and Rizk (44), in turn, characterize digital platforms as liminal governance environments in which experiences of support and control overlap. Another way that governance relates to mental health issues is via environmental and organizational regulation. Mir Mohamad Tabar et al. (36) discuss how water insecurity in Iran relates to mental health issues, which are linked not only by material conditions of scarcity but also by issues of poor governance that lead to more social conflicts and interpersonally based tensions. In an organizational context, Fox et al. (45) have identified that poor governance, specifically a lack of frameworks for managing risk of worker fatigue, relates to high occupational stress and poor psychological well-being. Crisis and emergency governance is another theme that appears repeatedly. Yildiz et al. (46) show in their study of the aftermath of the Turkey-Syria earthquake that the absence of children’s views from the process of crisis and emergency governance is related to poor psychological recovery outcomes. Other papers focus on the way in which legal and institutional systems impact mental health through recognition, protection, and care. The impact of limiting healthcare laws on transgender and non-binary youth, as reported by Carter et al. (47), and the insecurity and chronic stress caused by such laws, contribute to poor mental health outcomes, while laws and systems of migration, such as the Kafala system, as reported by Al Riachi and Diab (48), result in precarious mental health outcomes for migrant healthcare professionals. At the same time, a number of studies point out the role of governance in the protection of mental health. Le-Van and Bui (49), for example, establish that fair welfare governance structures in the Global South contribute to the mental health of mothers and children, indicating that the environment of governance is important in the psychological well-being of people. In general, this literature provides a narrative account of the significance of governance as a contextual mediator within ESG discourse on mental health. Unlike the literature that focuses on governance as a direct determinant of mental health, the literature reviewed here highlights its significance in influencing mental health contexts within ethical regulation, transparency, participation, accountability, and institutional protection. Quality of governance therefore stands out as a significant contextual factor that determines the impact of ESG forces on mental health outcomes. See Table 3.

**Table 3:** Narrative synthesis of governance dimensions linking ESG and mental health.

Narrative theme	Scope of the literature	Illustrative references (year)	Predominant study types	Recurring descriptive patterns in the literature	Interpretive relevance for mental health
Digital governance, AI, and ethical oversight	Governance of AI systems, digital mental health tools, algorithmic accountability, data ethics	Moseley (42); Hull (43); Gao et al. (25); Elyamany and Rizk (44); ICDEc(156); IntelliSys (157)	Policy analysis; qualitative institutional studies; case-based examinations of digital systems	The literature repeatedly describes tensions between care, surveillance, and trust in contexts where digital governance is opaque or weak	Mental health is narratively framed as sensitive to governance quality in digital environments, with transparency and ethical oversight discussed as supportive contexts for psychological safety
Governance of sustainability transitions and environmental resources	Eco-digital governance, environmental regulation, water and resource management	Zhang et al. (30); Mir Mohamad Tabar et al. (36)	Case studies; policy and institutional analyses; system-level assessments	Studies portray sustainability transitions as governance-dependent processes associated with both reduced uncertainty and heightened conflict	Mental health outcomes are interpreted as contingent on how governance structures manage environmental transitions, uncertainty, and resource-related stress
Organizational and institutional governance in health and crisis contexts	Workplace governance, healthcare regulation, disaster response, participatory decision-making	Fox et al. (45); Yildiz et al. (46)	Organizational case studies; crisis governance analyses; qualitative and mixed-method designs	The literature emphasizes how institutional practices and participation shape experiences of fatigue, burnout, and recovery	Mental well-being is discussed as embedded within organizational and crisis governance contexts that influence protection, recognition, and resilience
Legal, welfare, and migration governance	Welfare regimes, migration systems, legal frameworks, social protection policies	Al Riachi and Diab (48); Le-Van and Bui (49)	Legal and policy analysis; welfare and migration case studies	Studies describe contrasting mental health experiences across inclusive and exclusionary governance arrangements	Mental health is interpreted as shaped by legal and welfare contexts that regulate access to care, security, and social recognition

This table presents a descriptive and narrative synthesis of governance-related literature linking ESG and mental health. It organizes recurring themes and interpretive patterns without ranking evidence, assessing causal strength, or proposing formal governance models.

### 2.4 Narrative synthesis across environmental, social, and governance dimensions

With respect to the Environmental dimension, the literature reviewed reveals some regularities and significant differences. In general, environmental degradation and pollution are found to be related to negative outcomes in mental health, such as depression, anxiety, and stress disorders (50), while the results of cross-sectional studies are found to be more variable and dependent on specific contexts. The literature also presents a complex view of sustainability transitions, as there are findings related to favorable and unfavorable mental health experiences depending on the specific timing and population (51). Within the Social category, the importance of social inequality, job insecurity, housing, and the availability of social services is highlighted. At the same time, there is considerable diversity in the description of the associations with respect to the methods, welfare states, or groups. There is also the use of the life-course perspective or the model of cumulative disadvantage, which is often utilized to place the mental health process into context (52). By contrast, the body of literature focused on the Governance dimension is relatively more narrow in scope but also more cohesive in terms of narrative theme. Scholarship focused on institutional quality, rule of law, or social protection regimes typically conceives of governance as an intervening factor which conditions the experience of environmental and social risk (52). While differences in methodology, specifically in the measurement of governance indicators, are recognized, this body of literature typically conceives of governance as an influence on the conditions in which mental health risks and protections occur. Together, the literature appears to highlight a thematic convergence in relation to the importance of considerations of environment, society, and governance issues in the context of mental health issues and the continued state of fragmentation in relation to the approach and measures of comparison across different countries (50–52). Rather than cumulatively arriving at a conclusion or a final analytical approach in relation to the issues at hand, the literature and work highlighted the importance of a narrative approach in relation to the description and synthesis of the thematic patterns in relation to the environment, society, and governance factors. Therefore, the use of the empirical approach in the context of this work is to provide an approach in relation to the narrative synthesis of the literature.

## 3 Data sources and descriptive analytical approach to ESG and mental health

**Figure 1:**
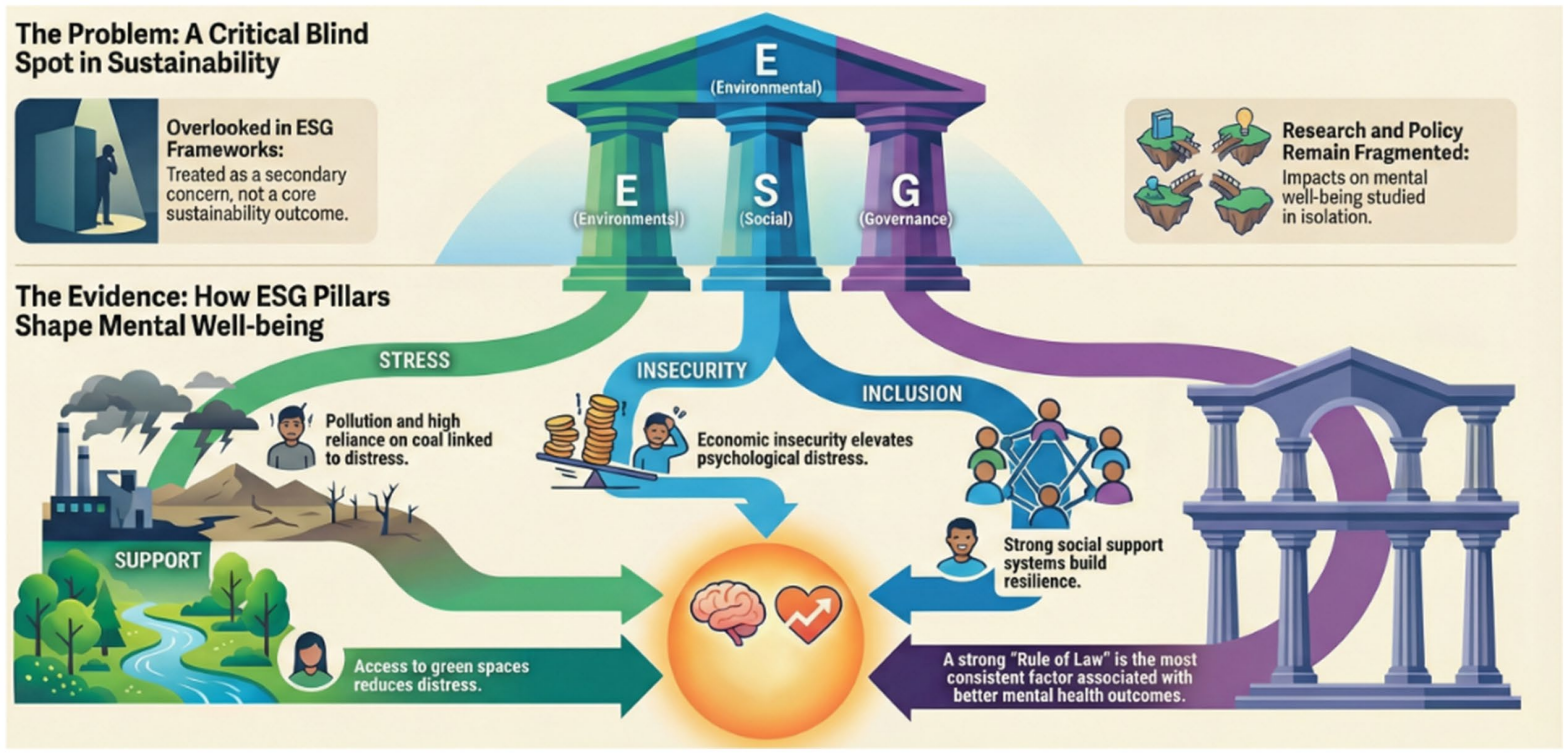
Descriptive overview of ESG pillars and their contextual associations with mental well-being. This figure provides a descriptive synthesis of how environmental, social, and governance dimensions are associated with population mental well-being. It illustrates contextual linkages—such as stress, insecurity, inclusion, and institutional support—without implying causal mechanisms or policy effectiveness.

The contextual information about environmental, social, and governance factors comes from the World Bank’s Sovereign ESG database. This database offers country-level indicators in a standardized form. These indicators are used as descriptive proxies to define sustainability environments in countries rather than behavioral decisions or optimized results (9). Environmental factors cover dimensions of energy use, environment efficiency, renewable energy, and environment protection. This database covers 31 countries between 2010 and 2022 with an unbalanced panel structure. Incomplete information in the database has been treated with interpolation and extrapolation methods to ensure smooth and transparent treatment of gaps in line with best practices in cross-country studies (53). These techniques are applied in an exploratory and descriptive fashion for data summarization purposes. Panel data statistical models are employed to address the issue of repeated observations within countries as well as the description of country differences across time, with a non-causal objective (in other words, there is no intention to identify structural parameters) (54). Standard techniques are applied for the internal validity and integrity of the descriptive analysis. Moreover, unsupervised machine learning algorithms are applied for the extraction of country clusters based on their mental health prevalence rates as well as their ESG factors. Various clustering algorithms such as k-means clustering, hierarchical clustering, fuzzy clustering, density-based clustering algorithms, as well as model-based clustering algorithms are applied for the extraction of country patterns based on their mental health data (55). These algorithms are applied for data pattern recognition purposes rather than prediction or causal analysis purposes. The validity of the clustering algorithms is evaluated based on internal quality criteria as well as Herfindahl–Hirschman Indices. Overall, the combination of data summarization algorithms with clustering algorithms provides a descriptive mapping of empirical patterns connecting mental health data with ESG factors across countries. This empirical part does not substitute the narrative review but rather aids the review by mapping observed patterns in a clear and exploratory fashion that is consistent with the descriptive and integrative nature of this research (see Figure 1).

### 3.1 Empirical strategy: descriptive and non-causal framework

The empirical strategy used in this study is exploratory and correlation analysis. Panel regression analysis and clustering analysis have been used to identify patterns of association, co-movements, and heterogeneities across countries between ESG variables and mental health issues. The analysis has no intention or aim at establishing a casual link between variables. The analysis does not involve an identification strategy that uses instruments, natural experiments, or policy shocks to identify exogenous variation. Therefore, the findings of this analysis can be interpreted for association and not effect.

## 4 Descriptive statistics and data completion for cross-country mental health trends

This section presents a descriptive explanation of data preparation and basic statistical profiling for understanding cross-country patterns of mental and behavioral disorders by using data from 2010 to 2022. Due to the nature of global time series data, it can be expected that some data points can be incomplete or missing. To make these points comparable across countries and years, missing data points were handled by transparent linear methods, which included interpolation for missing points in the observation series and extrapolation for points outside the observed range (56). With data harmonization, a full panel of 13 years for 32 countries was constructed for descriptive profiling purposes. Missing data points between two observed points were estimated by linear interpolation, which followed conventional procedures for descriptive time series analysis (57). Missing points outside the observed range were estimated by linear extrapolation based on trend conditions, which allowed for maintaining continuity and keeping procedures transparent (56). All these points were estimated and interpreted with care, being parts of an exploratory descriptive analysis. The constructed data set allows for a full descriptive characterization of mental health data across countries. Descriptive analysis of summary statistics has shown that there was substantial variation in mean values and measures of dispersion across countries, which highlighted differences in mental health profiles across countries (58). Some countries, namely Greece, Israel, Malta, Tajikistan, and Azerbaijan, were shown to have lower mean values, while Spain, Lithuania, Estonia, Slovakia, Latvia, and Czech Republic had relatively higher mean values during this analysis (58). Variability measures have further highlighted differences across countries (59). In some cases, there was relative stability in mental health values, while in other cases, there was more variability, which highlighted differences in mental health dynamics and reporting (59). Skewness and kurtosis measures have shown that there was no universality in mental health values across countries, which highlighted that there were episodic changes, structural changes, or other influences (60). Normality tests have been shown to provide further descriptive explanations regarding mental health distributions across countries, which highlighted mixed responses across countries (60). In some instances, the distribution of prevalence is close to symmetry, while in others, deviations from the normal distribution are found, in keeping with non-linear trends over time (59, 60). These features of the data are not considered to represent statistical tests of hypotheses, but are more a reflection of the heterogeneity of the observed distributions of prevalence. In general, the set of descriptive statistics included in this report point to significant cross-national differences in the level and variability of mental health prevalence. These trends are considered to represent the complex interplay of a range of factors, including the local socio-economic environment, the capacity of the health care system, cultural differences, and the practice of diagnostic criteria, and are not considered to represent uniform international trends (58). Such descriptive profiling serves as a backdrop for the following narrative and exploratory analyses (see Table 4).

**Table 4:** Descriptive statistics of mental health prevalence across 31 countries (2010–2022).

Statistics	ALB	ARM	AZE	BLR	BEL	BIH	BGR	CZE	DNK	EST	FIN	GEO	GRC	HUN	ISR	ITA
Valid	13	13	13	13	13	13	13	13	13	13	13	13	13	13	13	13
Missing	0	0	0	0	0	0	0	0	0	0	0	0	0	0	0	0
Mode	0.940	1.380	0.730	1.140	1.220	2.760	1.700	5.610	3.130	6.830	1.810	1.510	0.310	1.760	0.250	1.260
Median	1.210	1.720	1.000	1.140	1.220	3.160	1.780	5.630	3.160	6.970	1.760	2.170	0.320	1.700	0.300	1.280
Mean	1.134	1.747	1.046	1.133	1.216	3.151	1.810	5.664	3.192	6.980	1.762	2.054	0.327	1.672	0.301	1.305
Std. error of Mean	0.050	0.068	0.061	0.008	0.007	0.057	0.044	0.108	0.042	0.048	0.011	0.087	0.005	0.023	0.012	0.016
95% CI Mean upper	1.243	1.896	1.180	1.150	1.232	3.275	1.905	5.898	3.283	7.085	1.787	2.243	0.338	1.722	0.327	1.340
95% CI mean lower	1.025	1.598	0.913	1.117	1.200	3.026	1.715	5.429	3.100	6.875	1.738	1.865	0.316	1.621	0.275	1.269
Std. deviation	0.181	0.247	0.221	0.027	0.026	0.206	0.157	0.388	0.151	0.174	0.041	0.313	0.019	0.084	0.043	0.059
Coefficient of variation	0.159	0.141	0.211	0.024	0.021	0.065	0.087	0.069	0.047	0.025	0.023	0.152	0.058	0.050	0.142	0.045
MAD	0.120	0.210	0.160	0.020	0.010	0.080	0.130	0.190	0.110	0.110	0.030	0.150	0.010	0.060	0.040	0.040
MAD robust	0.178	0.311	0.237	0.030	0.015	0.119	0.193	0.282	0.163	0.163	0.044	0.222	0.015	0.089	0.059	0.059
IQR	0.320	0.410	0.370	0.030	0.020	0.160	0.280	0.310	0.220	0.230	0.070	0.520	0.020	0.120	0.070	0.070
Variance	0.033	0.061	0.049	7.397 × 10–4	6.756 × 10–4	0.042	0.025	0.151	0.023	0.030	0.002	0.098	3.564 × 10–4	0.007	0.002	0.003
Skewness	−0.334	0.077	0.376	−0.354	−0.475	−0.298	0.549	−0.890	−0.030	−0.043	−0.283	−0.717	1.227	−0.897	0.643	0.519
Std. error of skewness	0.616	0.616	0.616	0.616	0.616	0.616	0.616	0.616	0.616	0.616	0.616	0.616	0.616	0.616	0.616	0.616
Kurtosis	−1.717	−0.907	−0.966	−0.792	0.895	0.293	−1.249	1.976	−0.993	0.969	−1.181	−0.873	0.792	0.525	−0.141	−0.823
Std. error of kurtosis	1.191	1.191	1.191	1.191	1.191	1.191	1.191	1.191	1.191	1.191	1.191	1.191	1.191	1.191	1.191	1.191
Shapiro–Wilk	0.865	0.969	0.941	0.927	0.948	0.970	0.889	0.939	0.956	0.970	0.922	0.891	0.831	0.894	0.939	0.931
*p*-value of Shapiro–Wilk	0.044	0.876	0.469	0.308	0.575	0.894	0.093	0.444	0.689	0.889	0.269	0.101	0.016	0.112	0.440	0.351
Range	0.510	0.800	0.700	0.080	0.100	0.750	0.440	1.550	0.480	0.700	0.120	0.910	0.060	0.280	0.140	0.190
Minimum	0.860	1.380	0.730	1.090	1.160	2.760	1.630	4.730	2.930	6.630	1.690	1.510	0.310	1.480	0.250	1.220
Maximum	1.370	2.180	1.430	1.170	1.260	3.510	2.070	6.280	3.410	7.330	1.810	2.420	0.370	1.760	0.390	1.410
25th percentile	0.940	1.520	0.900	1.120	1.210	3.080	1.700	5.600	3.110	6.850	1.730	1.770	0.310	1.620	0.260	1.260
50th percentile	1.210	1.720	1.000	1.140	1.220	3.160	1.780	5.630	3.160	6.970	1.760	2.170	0.320	1.700	0.300	1.280
75th percentile	1.260	1.930	1.270	1.150	1.230	3.240	1.980	5.910	3.330	7.080	1.800	2.290	0.330	1.740	0.330	1.330
Sum	14.740	22.710	13.600	14.730	15.810	40.960	23.530	73.630	41.490	90.740	22.910	26.700	4.250	21.730	3.910	16.960

	KAZ	KGZ	LVA	LTU	MLT	POL	MDA	ROU	RUS	SMR	SVK	ESP	TJK	TKM	UKR	UZB
Valid	13	13	13	13	13	13	13	13	13	13	13	13	13	13	13	13
Missing	0	0	0	0	0	0	0	0	0	0	0	0	0	0	0	0
Mode	1.480	1.250	5.030	6.160	0.100	4.050	3.570	1.350	2.650	3.290	6.680	10.090	0.500	1.130	3.750	1.050
Median	1.940	1.250	5.990	9.460	0.250	4.120	4.300	2.140	2.720	3.330	7.060	11.640	0.520	1.180	3.740	1.050
Mean	2.182	1.242	5.991	9.417	0.260	4.167	4.285	2.082	2.754	3.531	7.073	11.738	0.516	1.242	3.828	1.062
Std. error of Mean	0.189	0.021	0.173	0.581	0.028	0.100	0.131	0.144	0.027	0.095	0.062	0.301	0.012	0.056	0.141	0.028
95% CI Mean upper	2.593	1.289	6.367	10.682	0.320	4.384	4.570	2.395	2.812	3.739	7.209	12.393	0.543	1.364	4.135	1.124
95% CI Mean lower	1.770	1.196	5.615	8.152	0.200	3.950	4.000	1.770	2.696	3.323	6.937	11.083	0.490	1.121	3.522	1.000
Std. Deviation	0.682	0.077	0.622	2.093	0.099	0.359	0.471	0.518	0.096	0.344	0.225	1.084	0.044	0.201	0.508	0.103
Coefficient of variation	0.312	0.062	0.104	0.222	0.382	0.086	0.110	0.249	0.035	0.097	0.032	0.092	0.085	0.162	0.133	0.097
MAD	0.350	0.050	0.480	1.740	0.060	0.130	0.200	0.240	0.080	0.060	0.160	0.980	0.020	0.110	0.380	0.050
MAD robust	0.519	0.074	0.712	2.580	0.089	0.193	0.297	0.356	0.119	0.089	0.237	1.453	0.030	0.163	0.563	0.074
IQR	0.830	0.070	0.960	4.080	0.110	0.270	0.310	0.690	0.170	0.580	0.310	1.780	0.040	0.250	0.980	0.090
Variance	0.465	0.006	0.387	4.382	0.010	0.129	0.222	0.268	0.009	0.118	0.051	1.175	0.002	0.041	0.258	0.011
Skewness	1.038	−1.641	0.003	−0.349	−0.175	1.148	1.012	0.192	0.158	0.910	0.299	−0.176	−0.149	0.560	0.388	0.632
Std. error of skewness	0.616	0.616	0.616	0.616	0.616	0.616	0.616	0.616	0.616	0.616	0.616	0.616	0.616	0.616	0.616	0.616
Kurtosis	0.104	3.312	−1.200	−1.284	−0.748	3.242	1.081	−0.544	−1.831	−0.902	−0.133	−1.688	−0.327	−0.467	−1.282	0.957
Std. Error of kurtosis	1.191	1.191	1.191	1.191	1.191	1.191	1.191	1.191	1.191	1.191	1.191	1.191	1.191	1.191	1.191	1.191
Shapiro–Wilk	0.873	0.842	0.966	0.915	0.948	0.886	0.880	0.940	0.883	0.781	0.973	0.896	0.977	0.941	0.877	0.966
*P*-value of Shapiro–Wilk	0.058	0.023	0.841	0.218	0.562	0.085	0.070	0.460	0.078	0.004	0.928	0.119	0.960	0.477	0.064	0.836
Range	2.070	0.280	1.920	6.230	0.310	1.440	1.680	1.650	0.260	0.920	0.820	2.990	0.150	0.660	1.430	0.390
Minimum	1.480	1.040	5.030	6.160	0.100	3.650	3.570	1.350	2.630	3.210	6.680	10.090	0.440	0.970	3.120	0.900
Maximum	3.550	1.320	6.950	12.390	0.410	5.090	5.250	3.000	2.890	4.130	7.500	13.080	0.590	1.630	4.550	1.290
25th percentile	1.690	1.230	5.510	7.090	0.210	4.050	4.030	1.630	2.680	3.290	6.910	10.840	0.500	1.130	3.480	1.000
50th percentile	1.940	1.250	5.990	9.460	0.250	4.120	4.300	2.140	2.720	3.330	7.060	11.640	0.520	1.180	3.740	1.050
75th percentile	2.520	1.300	6.470	11.170	0.320	4.320	4.340	2.320	2.850	3.870	7.220	12.620	0.540	1.380	4.460	1.090
Sum	28.360	16.150	77.880	122.420	3.380	54.170	55.710	27.070	35.800	45.900	91.950	152.590	6.710	16.150	49.770	13.800

This table offers a comprehensive view of the mental health prevalence statistics with 31 countries included, taking into consideration the measures of central tendency, dispersion, as well as the distribution of the statistics. This table points to the differences at the national level with regards to the reporting of the statistics.

**Figure 2:**
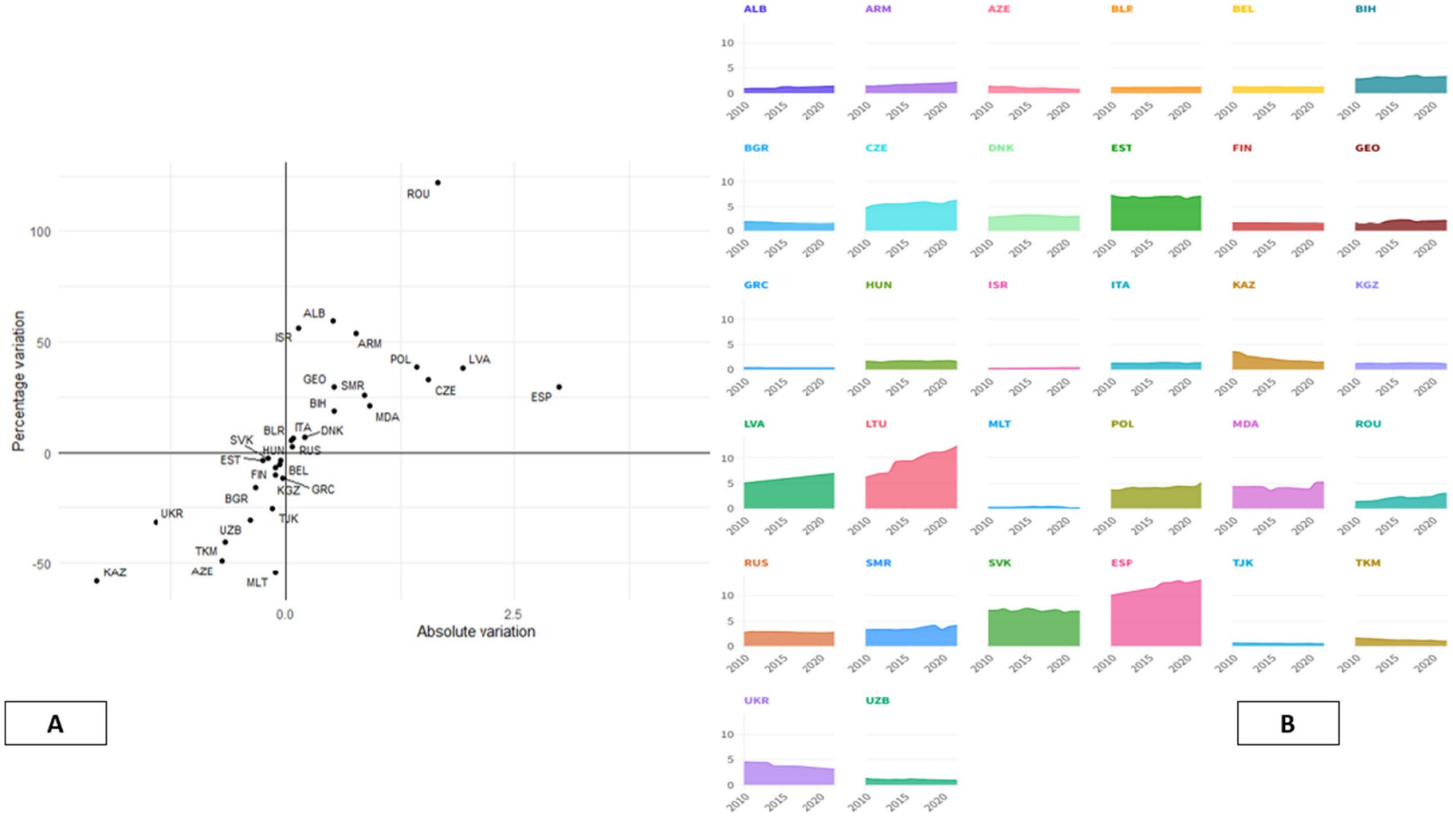
Cross-national trends in mental health disorder prevalence (2010–2022). The dual-panel graph provides information on the absolute and relative differences in the prevalence of mental health problems in countries **(A)**, while **(B)** shows the annual path of trends. Through the combination of summary information based on slopes and longitudinal trends, it is possible to observe differences in the prevalence of problems among countries.

The two panels provide a complementary descriptive graph on trends in the prevalence of mental and behavioral disorders across countries from 2010 to 2022. The graph combines a summary graph on trends with the respective time series trends, thereby presenting a comprehensive perspective on cross-country disparity in observed trends (61). The scatter diagram in Panel A uses the horizontal axis to represent absolute trends and the vertical axis to represent percentage changes. The aim here is to compare the trends by summarizing the direction and magnitude of the trends. The upper-right quadrant represents the positive absolute and percentage trends for the following countries: Romania, Lithuania, Latvia, Spain, Poland, the Czech Republic, Albania, and Armenia. The countries close to the origin, such as Finland, Denmark, and Russia, have less variation over time, indicating a stable pattern in the prevalence (62). The left quadrants represent the declining trends for the following countries: Kazakhstan, Azerbaijan, and Turkmenistan, which have declining trends in the prevalence over the period under observation (61). What helps to fill this gap is the use of line plots by Panel B to show the country-specific path of mental health prevalence. This helps the reader not only see the trend of the prevalence levels at different points in time but also see the differences between the trends of the various countries on the graph (63). Countries highlighted by Panel A as having positive variation, such as Spain, Lithuania, Latvia, Romania, and Poland, will generally show a trend that slopes upwards throughout the period, while countries with negative variation will show a trend that slopes downwards (64). Together, the two panels above demonstrate the viability of the descriptive analysis model. Panel A effectively captures the trend summary, while Panel B helps to demonstrate the time dynamics behind the summaries. It is clear from the graphical evidence that the patterns demonstrated by mental health prevalence rates vary from country to country, which could be the result of the differences in the various national contexts (62, 64). Additionally, the lack of synchronized breaks in the data further supports the fact that the trend summary is not consistent over time, thereby making the country-specific trend relevant (61, 63). See Figure 2.

## 5 Environmental sustainability and energy structures in relation to mental health patterns

This section describes the relationship between the Environmental (E) dimension of the ESG framework and the patterns found in the prevalence of mental health. Although the degradation of the environment has been well-established as having an influence on physical health, the relationship between the environment and mental well-being has been comparatively less well-explicated. Nonetheless, an increasing number of studies have found relationships between the environment, such as exposure to pollutants, the use of fossil fuels, environmental stress, and the pace of environmental change, and mental health, such as anxiety disorders and depressions. Using cross-national data from 31 countries from 2010 to 2022, this paper describes the alignment between the prevalence of mental health, as measured by the World Health Organization, and the environment, measured by the Sovereign ESG database from the World Bank. Although the variables included in the analysis, such as the use of coal-powered energy, energy intensity, energy use per capita, the use of renewable energy, and the extent of protected lands, could be included in an equation to model the relationship between the environment and mental well-being, the focus here is on the description, which enables the examination of differences across time and across countries. This quantitative analysis enables the examination of differences across time, providing an indication of the differences across countries, without implying any model or explanation. This description across countries, which enables the examination of differences, collectively establishes the co-occurrence between the indicators of mental health prevalence and the indicators of the environment. This description, which enables the examination across time, collectively establishes the co-occurrence between the indicators of mental health prevalence and the indicators of the environment, which is consistent with the narrative description of the Environmental dimension of the ESG framework (see Table 5).

**Table 5:** Definition of environmental (E) and mental health variables used in the analysis.

Variable	Code	Definition
Mental health prevalence	Mental	Measures the prevalence of mental disorders (%) in the population, based on WHO data. It represents the cumulative number of registered mental disorder cases at the end of each calendar year, classified under Chapter V of ICD-9/10, and reflects diagnosed mental health conditions within a country.
Electricity from coal	ELCL	Measures the share of total electricity production generated from coal-fired power plants. It captures a country’s reliance on fossil fuels within its energy mix and serves as a proxy for carbon intensity, air pollution exposure, and environmentally unsustainable energy production patterns.
Energy intensity	ENIN	Indicates the amount of energy consumed per unit of economic output, typically expressed as energy use relative to GDP. Lower values reflect higher energy efficiency, technological advancement, and reduced environmental pressure, while higher values signal inefficient energy use and greater ecological stress.
Energy use per capita	ENPC	Represents the average amount of energy consumed per person within a country. This indicator reflects living standards, consumption patterns, and energy accessibility, but also captures environmental pressure and lifestyle-related factors linked to resource use and sustainability.
Renewable electricity	RELE	Measures the proportion of electricity generated from renewable energy sources such as wind, solar, hydro, and biomass. It reflects the degree of transition toward sustainable energy systems and a country’s commitment to reducing greenhouse gas emissions and environmental degradation.
Protected areas	PROT	Represents the percentage of national territory designated as legally protected areas for biodiversity conservation and ecosystem preservation. This variable captures environmental governance quality, conservation efforts, and long-term commitment to environmental sustainability within the ESG framework.

This table summarizes the core dependent and environmental explanatory variables employed in the empirical analysis. All indicators are defined at the country level and sourced from internationally harmonized databases (WHO and World Bank), ensuring cross-country comparability within the ESG framework.

We have estimated the following equation:


$$\begin{array}{l} \text{Menta}l_{\mathrm{it}}=\alpha +\beta_{1}{\left(\text{ELCL}\right)}_{\mathrm{it}}+\beta_{2}{\left(\text{ENIN}\right)}_{\mathrm{it}}+\beta_{3}{\left(\text{ENPC}\right)}_{\mathrm{it}}\\ +\beta_{4}{\left(\text{RELE}\right)}_{\mathrm{it}}+\beta_{5}{\left(\text{PROT}\right)}_{\mathrm{it}} \end{array}$$

Where i = 31 and t = 13. See Table 6.

**Table 6:** Panel regression results: environmental (E) ESG indicators and mental health prevalence.

Observations	269
Included cross-sectional units	30

Time-series length	Minimum 8, maximum 9	
Models	Fixed-effects,	Random-effects (GLS) Using Nerlove’s transformation

Dependent variable	Mental					
	Coefficient	Std. error	t-ratio	Coefficient	Std. error	z
Const	−0.15	0.41	−0.38	−0.10	0.67	−0.15
ELCL	0.018***	0.005	3.51	0.017***	0.005	3.52
ENIM	−0.003***	0.001	−2.90	−0.003***	0.001	−2.7
ENPC	0.0004***	0.0001	4.36	0.0004***	0.0001	4.43
RELE	0.033***	0.005	6.22	0.032***	0.0052	6.303
PROT	0.048***	0.01	3.92	0.047***	0.011	4.03
Statistics	Mean dependent var	3.08		Mean dependent var	3.08	
	Sum squared resid	23.31		Sum squared resid	2046.85	
	LSDV R-squared	0.98		Log-likelihood	−654.64	
	LSDV *F*(34, 234)	650.28		Schwarz criterion	1342.85	
	Log-likelihood	−52.75		rho	0.46	
	Schwarz criterion	301.33		S.D. dependent var	2.88	
	rho	0.46		S.E. of regression	2.78	
	S.D. dependent var	2.88		Akaike criterion	1321.28	
	S.E. of regression	0.31		Hannan-Quinn	1329.94	
	Within R-squared	0.21		Durbin-Watson	0.73	
	P-value(F)	3.4e-212				
	Akaike criterion	175.51				
	Hannan-Quinn	226.04				
	Durbin-Watson	0.73				
Tests	Joint test on named regressors Test statistic: *F*(5, 234) = 13.17 with *p*-value = P(F(5, 234) > 13.1707) = 2.61455e-11			‘Between’ variance = 7.87 ‘Within’ variance = 0.086 mean theta = 0.96 Joint test on named regressors Asymptotic test statistic: Chi-square(5) = 68.80 with *p*-value = 1.81623e-13		
	Test for differing group intercepts Null hypothesis: The groups have a common intercept Test statistic: *F*(29, 234) = 661.03 with *p*-value = P(F(29, 234) > 661.031) = 2.24594e-207			Breusch-Pagan test Null hypothesis: Variance of the unit-specific error = 0 Asymptotic test statistic: Chi-square(1) = 1035.04 with *p*-value = 4.3547e-227		
	Distribution free Wald test for heteroskedasticity Null hypothesis: the units have a common error variance Asymptotic test statistic: Chi-square(30) = 8892.52 with *p*-value = 0			Hausman test Null hypothesis: GLS estimates are consistent Asymptotic test statistic: Chi-square(5) = 1.63281 with *p*-value = 0.897253		
	Test for normality of residual Null hypothesis: error is normally distributed Test statistic: Chi-square(2) = 294.57 with *p*-value = 1.08103e-64			Test for normality of residual Null hypothesis: error is normally distributed Test statistic: Chi-square(2) = 185.15 with p-value = 6.21338e-41		
	Wooldridge test for autocorrelation in panel data - Null hypothesis: No first-order autocorrelation (rho = −0.5) Test statistic: *F*(1, 29) = 68.60 with *p*-value = P(F(1, 29) > 68.60) = 3.9356e-09					
	Pesaran CD test for cross-sectional dependence Null hypothesis: No cross-sectional dependence Asymptotic test statistic: *z* = 0.83 with *p*-value = 0.40			Pesaran CD test for cross-sectional dependence Null hypothesis: No cross-sectional dependence Asymptotic test statistic: *z* = 0.84 with *p*-value = 0.39		

Note. The table reports panel estimates for 30 countries (269 observations, 2010–2022). The dependent variable is the WHO Prevalence of mental disorders (%) (HFA_391). Fixed-effects and random-effects (GLS) models with Nerlove’s transformation are used. Diagnostic tests indicate country heterogeneity and autocorrelation, no cross-sectional dependence, and a Hausman test supporting the random-effects specification. ***, **, * denote significance at 1, 5, and 10%.

The Environmental (E) component of ESG factors is generally defined by indicators that focus on energy production, use, efficiency, emission, and protection of the ecosystem (65). From this context, the choice of indicators for the environment focuses on capturing the sustainability context within which mental health outcomes are measured, rather than on the process that directly affects mental health outcomes. Thus, mental health outcomes are viewed within the context of broader sources of environmental pressure, exposure to stress, and sustainability transitions (66, 67). The current study connects mental health prevalence rates with a set of environmental variables—coal-based electricity (ELCL), energy intensity (ENIN), per capita energy use (ENPC), renewable electricity (RELE), and protected areas (PROT)—using cross-national data from 31 countries from 2010 to 2022. The current study uses a set of variables that work as a summary of a set of countries with different degrees of management and transition regarding their environmental management and transition challenges and difficulties, and it is an unbalanced panel with 269 observations. These techniques are used for exploratory as well as organizing purposes, allowing for the summarization of observed patterns across countries while considering repeated observations across time. This approach emphasizes heterogeneity with regard to environmental profiles as well as mental health prevalence across country contexts, neither structurally interpreting nor suggesting causal explanations (68). The results of the description show the co-occurrence of the variation in the mental health prevalence rate and the variation in the indicators of the environment and energy-related factors. This is because the high use of coal-powered energy is accompanied by the high level of mental health disorders’ prevalence rate as stated in the literature (67, 69). On the other hand, the use of energy and the energy efficiency rate is different for countries with different mental health conditions as stated in the literature (70). However, the patterns related to renewable energy sources and protected areas may be more complex. In some cases, the presence of countries with growing renewable energy sources or larger protected areas is also associated with high mental health prevalence. Instead of pointing to the presence of contradictory outcomes, the trends are examined by researchers as the result of the complexity of the transformation process related to institutions, social change, the inequitable distribution of benefits of the environment, or the awareness of mental health conditions during the transformation process (66, 71). In sum, there appears to be a complex intertwining of the ESG Environmental element and mental health outcomes, in ways which are likely to be non-linear and dependent on specific contexts. Poor environmental conditions, energy inefficiency, and consumption patterns tend to occur alongside high levels of mental health, while sustainable shifts may produce enabling and, at the same time, stressful conditions. Such findings seem to support the integration of mental health aspects into debates about environmental sustainability, while also pointing to the difficulties in using environmental metrics in isolation from their social and temporal contexts, as noted by Tosun in 2023 and by Hartinger et al. in 2024.

### 5.1 Environmental clustering and mental health: a k-means perspective

In this research, the use of the techniques of clustering is as exploratory tools in the organization and description of the patterns that arise out of the multi-dimensional data that is related to environmental, energy, and mental health indicators. In this case, the aim of the use of the techniques of clustering is not the identification of the optimal classification or predictive structure, but the demonstration of the differences and similarities among the country indicators. In order to fulfill this descriptive objective, several clustering methods are examined, including density-based, Fuzzy C-means, hierarchical, model-based, k-means, and random forest clustering. A number of clustering validity measures are discussed in order to describe the differences between the various clustering methods, rather than comparing these methods for their relative merits. More traditional measures of clustering validity, such as the Dunn Index and Calinski and Harabasz Index, are discussed alongside the Herfindahl–Hirschman Index (HHI), which is used in this study for descriptive purposes only (72, 73). Through all techniques, it is observed that a compromise between cluster separation, compactness, and balance is achieved. For example, hierarchical clustering provides a distinct and compact form of clustering, as it has a larger value of separation measures. Simultaneously, it has a larger value of HHI measures, indicating that a few clusters comprise a large number of observations. Descriptively, it has been observed that some of the clustering techniques are more concentrated on major patterns and may ignore smaller patterns that are less observed. In contrast to this, the density-based clustering method shows relatively equal distribution of cluster sizes, as indicated by the low HHI measures with relatively weaker distinction between the clusters. This scenario offers another dimension of description where equality among the groups is considered irrespective of the distinction among the clusters. The other techniques like random forest, model-based, and fuzzy c-mean clustering offer relatively equal measures of the two dimensions. Within this framework of comparative description, the k-means clustering method provides a balanced view of the data, with a level of separation that is neither too high nor too low and a fairly equal distribution of points in each cluster, which is a good balance according to Mallik et al. (74). This pattern is not seen as an optimal solution but rather a pragmatic descriptor that provides a framework for a comparative analysis of similarities and differences between countries. In general, the comparison of the methods for clustering emphasizes the fact that no technique is uniquely capable of identifying the underlying structure inherent in the data. Rather, the results from the clustering technique have been viewed as complementary descriptive summaries to help inform the organization of complex data, facilitating the narrative analysis of diversity across the countries (see Table 7).

**Table 7:** Comparative performance of clustering algorithms based on normalized validity indices.

Indicator	Density based	Fuzzy C-Means	Hierarchical	Model based	K-Means	Random forest
Maximum diameter	0.846	0.577	0.000	1.000	0.114	0.640
Minimum separation	0.225	0.000	1.000	0.917	0.285	0.917
Pearson’s γ	0.206	0.282	1.000	0.000	0.396	0.303
Dunn index	0.071	0.000	1.000	0.349	0.216	0.422
Entropy	1.000	0.428	0.121	0.000	0.376	0.349
Calinski–Harabasz index	0.391	0.262	0.826	0.000	1.000	0.535
HH Index	0.000	0.316	1.000	0.764	0.223	0.240

This table reports normalized (0–1) cluster validity metrics used to compare alternative clustering algorithms. Higher values indicate better performance for separation, cohesion, and structural quality, while lower values indicate preferable outcomes for dispersion and entropy-related measures. The results highlight the relative strengths of hierarchical clustering across multiple validity criteria.

This section presents a descriptive interpretation of the results from the k-Means clustering analysis, used as an exploratory method for summarizing the diversity in the dataset for various indicators related to the environment, energy, and mental health. The results from the clustering analysis are not considered an optimal classification but rather an exploratory way to compare the results through narrative approaches for each country profile (75). The k-Means algorithm identifies ten clusters that have vastly varying sizes, from very small groups such as Cluster 8 with two observations and Clusters 5 and 7 with eight each to larger groups such as Cluster 6 (with 50 observations), Cluster 1 (with 33), Cluster 10 (with 31), and Cluster 3 (with 29). The varying sizes of the clusters represent the data distribution rather than the concentration in a dominant cluster, as indicated by the low concentration of cluster sizes for k-Means clustering approaches using the Herfindahl–Hirschman Index (76). The sizes of the clusters also have varying levels of within-cluster variability. Medium-to-large clusters such as Clusters 3 and 6 have a larger share in the overall variability, whereas smaller clusters have less because they have fewer observations (77). Descriptively, the pattern shows the existence of cross-country profiles and niche configurations within the dataset. The silhouette values also demonstrate the varying cluster structures. Clusters such as Clusters 5, 7, and 8 have high silhouette values, indicating they have clearly defined groups, while the larger groups have moderate values, indicating they have varying degrees of homogeneity. Medium groups such as Cluster 9 have clearly defined groups as well (78). The cluster centers demonstrate clearly descriptive patterns for mental health, environment, and energy indicators. For example, Cluster 9 has comparatively higher values for the mental health indicator, while Clusters 2, 3, and 4 have lower values compared to the means (79, 80). Characteristics related to the environment and energy sources such as fossil fuel reliance, renewable energy variables, as well as variables related to environmental protection are also different across the clusters, leading to a distinct profile with a combination of contexts related to sustainability and the prevalence of mental health. Overall, the k-Means clustering technique has led to a descriptive mapping of country profiles that show the co-variation of mental health prevalence with environmental factors as well as energy patterns across varied contexts. The above-mentioned clusters are not taken as causal regimes but as constructs that facilitate the narrative exploration of heterogeneity across countries (see Table 8).

**Table 8:** Hierarchical clustering results and standardized cluster centers for environmental–mental health indicators.

Cluster	1	2	3	4	5	6	7	8	9	10
Size	33	16	29	24	8	50	8	2	16	31
Explained proportion within-cluster heterogeneity	0.136	0.109	0.265	0.100	0.011	0.216	0.008	0.005	0.041	0.109
Within sum of squares	281.392	224.870	546.585	205.595	21.792	444.597	17.394	11.077	83.672	225.408
Silhouette score	0.234	0.306	0.148	0.276	0.721	0.177	0.685	0.713	0.360	0.215
Center Mental	0.731	−0.769	−0.503	−0.902	−0.177	−0.295	−0.446	−0.739	2.148	0.363
Center ACFT	0.461	−0.343	−2.069	0.461	0.420	0.280	−0.082	0.453	0.461	0.461
Center ASNR	−0.472	2.467	0.041	−0.493	1.237	−0.384	1.814	1.754	−0.513	−0.443
Center ASFD	−0.293	−0.309	1.044	−0.267	−0.309	0.072	−0.309	−0.304	−0.309	−0.076
Center AGLD	−0.610	0.647	−0.341	−0.410	−1.928	0.702	1.916	1.821	−0.038	−0.275
Center AGVA	−0.602	1.568	0.928	−0.643	−0.440	0.388	−0.271	0.757	−0.640	−0.700
Center FWWD	−0.270	0.925	−0.251	0.036	−0.289	−0.125	−0.114	9.678	−0.214	−0.192
Center CO2P	−0.101	−0.769	−0.962	0.200	1.855	−0.607	1.983	1.249	−0.057	1.187
Center CDD	−0.821	1.678	−0.420	1.426	−0.768	−0.146	0.610	3.124	0.044	−0.652
Center ELCL	−0.413	−0.779	−0.021	0.244	−0.253	−0.397	1.847	−0.871	−0.395	1.160
Center ENIM	0.275	−2.109	0.226	0.610	−1.337	0.408	−1.914	−3.132	0.525	0.225
Center ENIN	−0.569	0.922	0.298	−1.052	1.910	0.127	1.024	3.024	−0.622	−0.171
Center ENPC	0.648	−0.898	−1.149	−0.162	1.810	−0.507	0.798	0.653	0.204	0.970
Center FERT	−0.396	0.694	1.107	0.110	−0.320	−0.402	1.638	1.690	−0.776	−0.458
Center FOSS	−1.333	1.479	−0.350	0.725	0.855	−0.050	1.484	1.569	−0.676	0.147
Center HI35	−0.205	0.946	−0.170	0.016	−0.199	−0.205	−0.029	8.915	−0.204	−0.205
Center HDD	0.365	−0.700	0.909	−1.352	2.737	−0.138	0.586	−1.120	−0.592	−0.089
Center LST	−0.883	1.415	−0.353	1.264	−2.644	0.120	0.023	2.342	0.388	−0.307
Center WSTR	−0.644	1.997	−0.012	0.537	−0.765	−0.411	0.003	3.036	−0.257	0.046
Center CH4P	−0.157	0.096	−0.234	−0.256	1.320	−0.183	1.093	9.472	−0.302	−0.248
Center N2OP	1.460	−0.436	−0.691	−0.769	−0.492	0.122	−0.106	0.666	−0.586	0.130
Center PM25	−1.036	1.275	1.329	−0.163	−0.718	0.090	0.349	0.256	−0.541	−0.460
Center RELE	0.918	−0.825	1.588	−0.296	−0.533	−0.497	−0.804	−1.189	−0.015	−0.578
Center RENC	1.465	−1.288	0.998	−0.429	−1.161	−0.283	−1.286	−1.413	−0.238	−0.194
Center SPEI	0.324	−0.291	0.590	−0.139	0.368	−0.243	0.070	−0.267	−0.213	−0.234
Center PROT	0.268	−1.038	−0.592	−0.433	−0.699	−0.045	−1.079	−1.191	1.189	1.134
Center TREE	−0.136	−0.222	−0.222	−0.209	4.963	−0.193	−0.221	−0.222	−0.166	−0.183

This section offers a qualitative explanation of the k-Means clustering outcome in relation to the co-occurrence of the prevalence of mental health conditions and environment factors in the ESG framework on different country profiles. This result shows various non-homogeneous formations of the co-occurrence of mental health conditions with different environment factors without assuming linearity between the factors (81). In the result, the coordinates of the cluster centers are in standard deviation units from the mean. From a mental health perspective, there are a number of clusters—namely Clusters 1, 4, 8, 9, and 10—that show a positive value for the mental health measure, which is a reflection that there is a greater than average level of mental health issues within each cluster. It is significant to note that each of these mental health clusters has a distinctly different environment, which shows that there is a wide range of environments within which there is a greater level of mental health issues reported (66). For instance, mental health values are high within Cluster 9 while there is a moderate level of environmental pressure within this cluster. In mental health studies, such a phenomenon is often highlighted with regard to factors such as the level of mental health diagnosis within a particular environment rather than environmental pressures (82). On the other hand, Cluster 8 has strong mental health values, together with very high scores for several indicators of environmental pressure, namely, freshwater withdrawal, agricultural value added, methane emissions, energy intensity, and per capita energy use, together with a moderate level of reliance on coal-based power generation. Such a pattern can be said to have a very good descriptive fit for places where high resource use and strong environmental pressure co-exist with high observed mental health prevalence (83). Clusters showing negative mental health values, for example, Clusters 2, 3, and 7, have another set of descriptive patterns altogether. In these, there is a tendency for high resource use, agricultural expansion, emissions, and fossil fuel use, which co-exist with lower observed mental health prevalence. In this case, Cluster 7 has high agricultural land use, high water withdrawal, and high carbon and methane emissions, although its mental health measure stays close to or slightly below average. In the literature, these patterns have been interpreted in terms of differences in mental health reporting, access, and diagnosis, and do not necessarily mean that mental health conditions are less prevalent (84). Similarly, Cluster 3 has very high forest resource use, and lower mental health values, showing another pattern wherein mental health and resource use do not have a strong positive association (85). Indicators related to energy add to the differentiation of profiles among clusters. Inequalities in the use of coal-fired electricity and fossil fuels, as well as renewable energy sources, are not evenly distributed among clusters with greater as well as smaller rates of mental illness. Rather than pointing to a clear correspondence, such findings are interpreted in the literature as capturing “complexities of sustainability transitions, where transformations of energy systems may overlap with transformations of awareness, capacity, or reporting of mental illness” (84, 85). Other indicators of environmental stresses such as cooling/heating degree days, land surface temperature, water stress, and exposure to particulate matter 2.5 further enhance the descriptive definition of the clusters. Clusters which suffer from extreme climate-related stresses, such as Clusters 5 and 8, are smaller but more homogeneous, thus showing how climate extremes are usually linked with specific environmental-mental health patterns instead of linear trends (66). Generally, the outcome of the k-Means clustering analysis highlights the non-linear and complex nature of the relationship between the prevalence of mental health and the Environmental dimension of ESG variables. The profiles revealed that the prevalence of mental health does not systematically correspond to either positive or negative conditions, but rather occurs through a variety of settings. This supports a narrative approach to the Environmental dimension, which focuses on variability and complexity rather than direct linear associations (81, 82). See Table 9.

**Table 9:** Cluster centers for standardized ESG and mental health indicators.

Cluster	Mental	ACFT	ASNR	ASFD	AGLD	AGVA	FWWD	CO2P	CDD	ELCL	ENIM	ENIN	ENPC
1	0.461	−0.61	−0.602	−0.293	−0.472	−0.821	−0.157	−0.101	−0.413	0.275	−0.569	0.648	−0.396
2	−0.343	0.647	1,568	−0.309	2,467	1,678	0.096	−0.769	−0.779	−2,109	0.922	−0.898	0.694
3	−2069	−0.341	0.928	1,044	0.041	−0.42	−0.234	−0.962	−0.021	0.226	0.298	−1,149	1,107
4	0.461	−0.41	−0.643	−0.267	−0.493	1,426	−0.256	0.2	0.244	0.61	−1,052	−0.162	0.11
5	0.42	−1928	−0.44	−0.309	1,237	−0.768	1,320	1855	−0.253	−1,337	1910	1810	−0.32
6	0.28	0.702	0.388	0.072	−0.384	−0.146	−0.183	−0.607	−0.397	0.408	0.127	−0.507	−0.402
7	−0.082	1916	−0.271	−0.309	1814	0.61	1,093	1983	1847	−1914	1,024	0.798	1,638
8	0.453	1821	0.757	−0.304	1754	3,124	9,472	1,249	−0.871	−3,132	3,024	0.653	1,690
9	0.461	−0.038	−0.64	−0.309	−0.513	0.044	−0.302	−0.057	−0.395	0.525	−0.622	0.204	−0.776
10	0.461	−0.275	−0.7	−0.076	−0.443	−0.652	−0.248	1,187	1,160	0.225	−0.171	0.97	−0.458

FERT	FOSS	HI35	HDD	LST	WSTR	CH4P	N2OP	PM25	RELE	RENC	SPEI	PROT	TREE
−1,333	−0.27	0.365	−0.205	−0.883	0.731	1,460	−1,036	0.268	0.918	1,465	0.324	−0.136	−0.644
1,479	0.925	−0.7	0.946	1,415	−0.769	−0.436	1,275	−1,038	−0.825	−1,288	−0.291	−0.222	1997
−0.35	−0.251	0.909	−0.17	−0.353	−0.503	−0.691	1,329	−0.592	1,588	0.998	0.59	−0.222	−0.012
0.725	0.036	−1,352	0.016	1,264	−0.902	−0.769	−0.163	−0.433	−0.296	−0.429	−0.139	−0.209	0.537
0.855	−0.289	2,737	−0.199	−2,644	−0.177	−0.492	−0.718	−0.699	−0.533	−1,161	0.368	4,963	−0.765
−0.05	−0.125	−0.138	−0.205	0.12	−0.295	0.122	0.09	−0.045	−0.497	−0.283	−0.243	−0.193	−0.411
1,484	−0.114	0.586	−0.029	0.023	−0.446	−0.106	0.349	−1,079	−0.804	−1,286	0.07	−0.221	0.003
1,569	9,678	−1,120	8,915	2,342	−0.739	0.666	0.256	−1,191	−1,189	−1,413	−0.267	−0.222	3,036
−0.676	−0.214	−0.592	−0.204	0.388	2,148	−0.586	−0.541	1,189	−0.015	−0.238	−0.213	−0.166	−0.257
0.147	−0.192	−0.089	−0.205	−0.307	0.363	0.13	−0.46	1,134	−0.578	−0.194	−0.234	−0.183	0.046

The following table provides the results of the k-means clustering in terms of the centroids of the ten country groups, and the variables include the prevalence of mental health, as well as some selected environmental, social, and governance (ESG) factors. These values represent the standardized within-cluster means, and the table is a way of describing the co-occurrence of the prevalence of mental health and the different characteristics of the countries in terms of the selected factors, among the different country groups.

**Figure 3:**
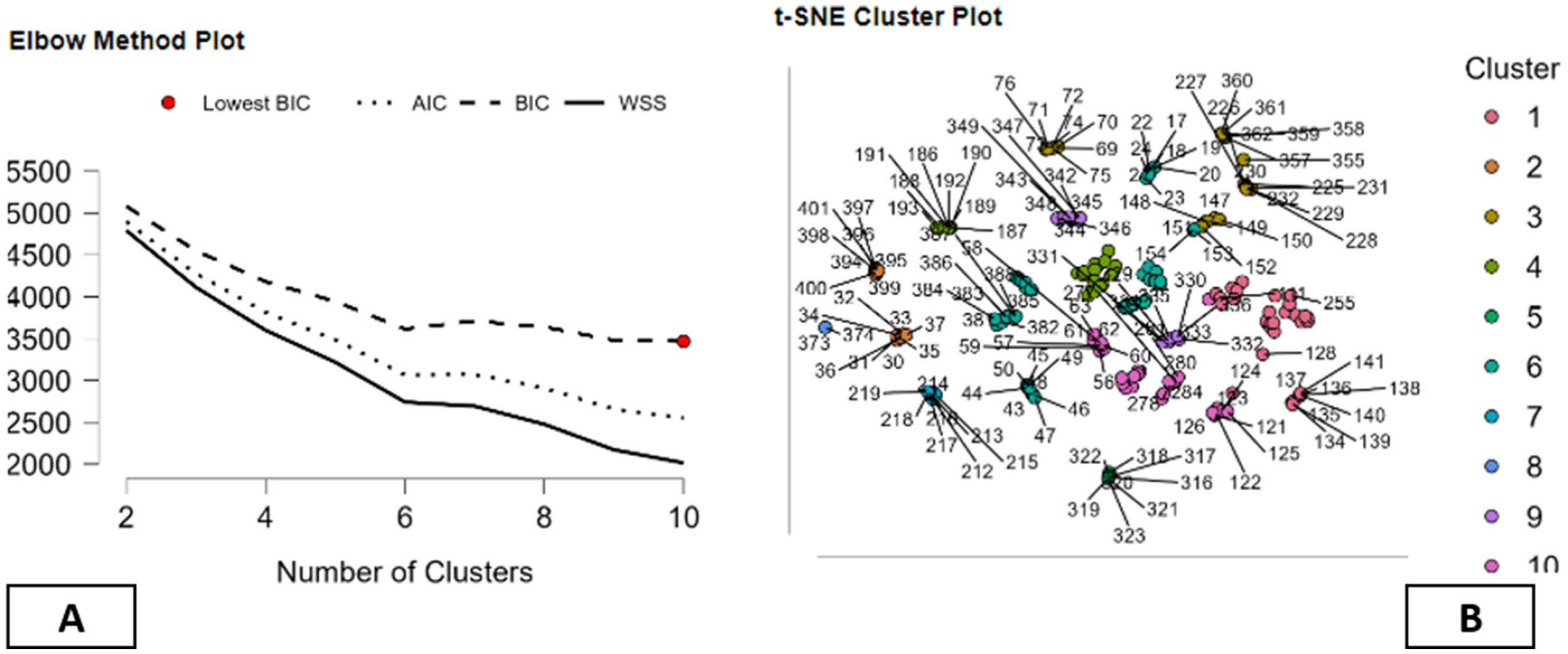
Model selection and cluster validation using elbow method and t-SNE visualization. The figure illustrates the selection and validation of *k*-means clustering. **(A)** Identifies the optimal number of clusters (*k* = 10) using the elbow method and information criteria (AIC, BIC). **(B)** Uses t-SNE visualization to show distinct, well-separated clusters, validating the clustering structure and capturing underlying data complexity.

The following two figures illustrate the procedure that was adopted to determine the k-Means clustering solution. Figure 3A shows the graph of the Elbow Method that defines the nature of intra-group variability in terms of the Within Sum of Squares (WSS). With increasing values of k, there is a progressive decrease in the values of WSS. This shows that intra-group variability is decreasing with increasing values of k. However, it can be noted that the rate of decrease in WSS is not constant. There is a point of diminishing return in the values of k approximately between *k* = 8 to *k* = 10, after which the values of k result in marginal improvements in intra-group variability (86). At the same time, the information criterion values of AIC and BIC in Figure A are progressively decreasing with increasing complexity of the model. Both AIC and BIC values are smoothly decreasing with increasing values of k. At the same time, AIC values are more sensitive to increasing complexity of the model than BIC values. Figure 3B serves as a complement to this data by offering a two-dimensional t-SNE visualization of the clustering result. Such a visualization provides an insightful way of understanding the distribution of data points in clusters when projected into a two-dimensional space in a way that maintains the local structure. In this visualization, the ten clusters are shown as more or less separate groups, some of which form more compact clouds, while others take more dispersed forms. Such differences in form can be attributed to the level of heterogeneity in the clusters, which corresponds with the level of within-cluster variability measured in the descriptive statistics for the clusters, as indicated by the silhouette statistics. However, it should be noted in this visualization that no individual cluster overshadows the whole space in the two-dimensional projection. Rather, the data points are dispersed in several clusters, showing a fairly balanced distribution. Where there are overlaps between clusters, such overlaps are minimal. Overall, while Figure 3A is a narrative description of the process by which cluster-fit values change as a function of cluster numbers, Figure 3B is a narrative description of the distribution of the formed clusters along the reduced dimensions. These two figures can therefore facilitate a comprehensive description of the k-Means clustering solution obtained.

The overall results show patterns of co-occurrences between the environmental and energy-related factors and the presence of mental health issues in varying degrees across different countries. Regardless of the specification of the alternative models, the patterns of association show similarities in the way the Environmental aspect of the ESG is consistently embedded in the mental health patterns of the cross-country settings. At the same time, the results require careful consideration and attention to the implications of the presence of high levels of mental health issues being reported in conjunction with other environmentally positive factors such as the use of renewable energy and the level of protected areas. These results may better be seen as an indication of overall capacity and level of awareness and reporting in the context of the sustainability transitions and the level of mental health issues rather than as an indication of the direct impact of the environment on mental health issues. At the same time, the use of aggregated variables in the analysis of the environment restricts the level of description of the analysis at the macro-level and prevents the identification of the underlying mechanisms and pathways. Overall, the results provide an indication of the overall complexity and non-linear relationship between the environment and mental health issues in the context of which the presence of both negative and positive aspects of the environment may be related to the presence of mental health issues in varying ways.

## 6 Social sustainability and mental health: descriptive evidence from the S pillar of ESG

This paper explores the structure of the empirical framework used to analyze the way in which the key social indicators related to the Social (S) aspect of the ESG framework match the trends for mental health prevalence. In recognizing the fact that mental health, as a matter of public health concern, exists within the context of the processes for social sustainability, the paper uses a set of variables related to socioeconomic, demographic, and infrastructure factors as a means for understanding the context within which the trends for mental health occur. Specifically, variables such as the Labor Force Participation Rate (LFPR), Under Five Mortality Rate (U5MR), Net Migration (NMIG), and Access to Safely Managed Drinking Water (WATS) have been used as indicators for the social development and capacity-building processes related to social inclusion. Taken as a whole, the variables serve as a descriptive indicator for the process related to social sustainability, covering the conditions for employment, the conditions for early childhood, the demographic processes, and the availability for essential social services. The paper uses the Fixed-Effects and Random-Effects model as an organizing framework for the repeated and cross-sectional data for the trends related to the manifestation of mental health prevalence for the varying conditions for the social context (see Table 10). The Social aspect of the ESG paradigm serves as a descriptive framework for the variables related to the constructs for mental health trends.

**Table 10:** Key socioeconomic and demographic indicators relevant to mental health and social sustainability.

Acronym	Indicator name	Short description
Mental	Mental health prevalence	Share of the population experiencing diagnosed or reported mental health conditions, reflecting both underlying psychosocial stress and the capacity of health systems to identify and record mental disorders.
LFPR	Labor force participation rate, total (% of population ages 15–64) (modeled ILO estimate)	Degree of social and economic inclusion of the working-age population, capturing access to employment, income opportunities, and exposure to work-related social pressures.
U5MR	Mortality rate, under-5 (per 1,000 live births)	Indicator of early-life health conditions and social protection, reflecting the quality of healthcare systems, nutrition, and broader social development.
NMIG	Net migration	Balance between immigration and emigration flows, capturing demographic change, social mobility, and pressures on social cohesion and integration mechanisms.
WATS	People using safely managed drinking water services (% of population)	Measure of access to essential basic services and social infrastructure, reflecting living standards, public health capacity, and social equity.

The above table describes the key socioeconomic factors, as well as demographic factors, which have been used to identify the environment in which the mental health status of the population is measured. Such factors as participation in the labor force, child mortality rates, net migration, and access to safe drinking water relate to the various aspects of mental health.

Specifically we have estimated the following equation:


$$\begin{array}{l} \text{Menta}l_{\mathrm{it}}=\alpha +\beta_{1}{\left(\text{LFPR}\right)}_{\mathrm{it}}+\beta_{2}{\left(U5\mathrm{MR}\right)}_{\mathrm{it}}\\ +\beta_{3}{\left(\text{NMIG}\right)}_{\mathrm{it}}+\beta_{4}{\left(\text{WATS}\right)}_{\mathrm{it}} \end{array}$$

Where i = 31 and t = 2010–2022. See Table 11.

**Table 11:** Fixed and random effects panel regression: social determinants and mental health prevalence.

Variables, statistics and metrics	Fixed-effects, using 401 observations Included 31 cross-sectional units Time-series length: minimum 12, maximum 13 Dependent variable: Mental			Random-effects (GLS), using 401 observations Using Nerlove’s transformation Included 31 cross-sectional units Time-series length: minimum 12, maximum 13 Dependent variable: Mental		
	Coefficient	Std. error	t-ratio	Coefficient	Std. error	z
const	−4.52023***	1.36161	−3.320	−4.55900***	1.42000	−3.211
LFPR	0.0484441***	0.0107110	4.523	0.0498748***	0.0105859	4.711
U5MR	0.0520566***	0.0152041	3.424	0.0485478***	0.0148387	3.272
NMIG	2.14448e-06***	3.36514e-07	6.373	2.13140e-06***	3.34832e-07	6.366
WATS	0.0409658***	0.0123354	3.321	0.04068***	0.0119559	3.403
Statistics	Mean dependent var	2.981820		Mean dependent var	2.981820	
	Sum squared resid	76.83029		Sum squared resid	2865.909	
	LSDV R-squared	0.975430		Log-likelihood	−963.3136	
	LSDV *F*(34, 366)	427.3509		Schwarz criterion	1956.597	
	Log-likelihood	−237.6957		rho	0.815926	
	Schwarz criterion	685.1800		S.D. dependent var	2.795951	
	rho	0.815926		S.E. of regression	2.686804	
	S.D. dependent var	2.795951		Akaike criterion	1936.627	
	S.E. of regression	0.458169		Hannan-Quinn	1944.535	
	Within R-squared	0.184508		Durbin-Watson	0.271810	
	P-value(F)	2.9e-272				
	Akaike criterion	545.3914				
	Hannan-Quinn	600.7440				
	Durbin-Watson	0.271810				
Tests	Joint test on named regressors Test statistic: *F*(4, 366) = 20.7022 with *p*-value = P(F(4, 366) > 20.7022) = 2.14262e-15			‘Between’ variance = 7.22267 ‘Within’ variance = 0.191597 mean theta = 0.954755 Joint test on named regressors - Asymptotic test statistic: Chi-square(4) = 85.6462 with *p*-value = 1.10628e-17		
	Test for differing group intercepts Null hypothesis: The groups have a common intercept Test statistic: *F*(30, 366) = 404.29 with *p*-value = P[F(30, 366) > 404.29] = 1.63411e-260			Breusch-Pagan test Null hypothesis: Variance of the unit-specific error = 0 Asymptotic test statistic: Chi-square(1) = 2239.57 with *p*-value = 0		
	Distribution free Wald test for heteroskedasticity Null hypothesis: the units have a common error variance Asymptotic test statistic: Chi-square(31) = 550,476 with *p*-value = 0			Hausman test Null hypothesis: GLS estimates are consistent Asymptotic test statistic: Chi-square(4) = 2.6501 with *p*-value = 0.617974		
	Test for normality of residual Null hypothesis: error is normally distributed Test statistic: Chi-square(2) = 436.088 with *p*-value = 2.01672e-95			Test for normality of residual Null hypothesis: error is normally distributed Test statistic: Chi-square(2) = 281.455 with *p*-value = 7.63659e-62		
	Wooldridge test for autocorrelation in panel data Null hypothesis: No first-order autocorrelation (rho = −0.5) Test statistic: *F*(1, 30) = 84.0531 with *p*-value = P(F(1, 30) > 84.0531) = 3.32295e-10					
	Pesaran CD test for cross-sectional dependence Null hypothesis: No cross-sectional dependence Asymptotic test statistic: *z* = 0.799721 with *p*-value = 0.423873			Pesaran CD test for cross-sectional dependence Null hypothesis: No cross-sectional dependence Asymptotic test statistic: *z* = 1.02254 with *p*-value = 0.306526		

This table presents fixed-effects and random-effects panel regression results examining the influence of key social indicators—labor participation, child mortality, net migration, and access to safe water—on mental health prevalence across 31 countries. Significant associations suggest that social inclusion, early-life health, and basic services are crucial to mental well-being.

These empirical findings can be made more interpretable by locating them firmly within the Social (S) component of the ESG approach, in which the prevalence of mental health is viewed as a socially embedded process, as opposed to a strictly defined clinical outcome (87, 88). In this way, the prevalence of mental health can be viewed as a measure of not merely underlying levels of psychosocial stress, but also as a function of the ability of societies to detect and record levels of mental health through their social and healthcare infrastructure. In this way, mental health outcomes are considered to be a social outcome, reflecting the level of social integration, resilience to adversity, and functionality of social infrastructure and protective mechanisms. The social indicators used for analysis, which include the labor force participation rate (LFPR), the under-five mortality rate (U5MR), net migration (NMIG), and access to safely managed drinking water services (WATS), are informed by their use in defining the essential facets of social sustainability. Taken together, these indicators encompass aspects of labor market integration, early life health outcomes, demography, and access to basic services, which remain core pillars of social development and social equity (55, 89). Contrary to their use as direct predictors of outcomes related to mental health, these indicators are used as a means of defining the social contexts within which the prevalence of mental health outcomes exist. Through a panel data set of 31 countries between 2010 and 2022, this study examines how mental health prevalence rates differ across different social settings and over different trajectories. The fixed effects and random effects models are essentially a device that organizes observations over time across countries, taking into account cross-national differences that persist over time. Within this organizational device, findings highlight that mental health prevalence rates differ markedly across different countries, indicating that mental health is intricately situated within existing social structures that are relatively static and cannot be adequately represented by a single indicator (90, 91). As shown in Table 11, the tests also confirm the significant cross-country heterogeneity indicated by the rejection of the common intercepts hypothesis. This also confirms the idea that cross-country differences in mental health prevalence are influenced by fundamental social structures, which can be equated to the structural components of the Social aspect of ESG factors. These factors are difficult to quantify accurately but play a crucial part in determining mental health experiences in societies. They can also be equated to the non-structural components of the Social aspect of ESG factors. In this light, the Hausman test reveals that the random effects model offers a consistent explanation of these relationships in the data, implying that these unobserved country-specific factors are not systemically correlated with the social indicators that are being accounted for. This finding does not point toward any causal relationships between these factors but rather lends further support to the view that these identified factors are merely good proxies for overall social sustainability factors in the ESG framework in studies that concern themselves with the social aspects of ESG considerations. Out of all the social indicators reviewed, the involvement in the labor force always corresponds with increased reported mental health prevalence. However, it must be noted that the positive correlation should not be interpreted as suggesting that increased engagement in the labor market corresponds with worse mental health outcomes. Rather, the increased engagement in the labor market may be reflective of increased social inclusion, increased institutional capacity, and advanced systems for the detection and recording of mental health problems (92, 93). In other words, in the presence of increased engagement in the labor market for the working-age population, there may be increased interaction with institutional frameworks such as the workplace, the healthcare sector, and social security systems that enable the recording of mental health problems. Likewise, the under-five mortality rate is another indicator of early life conditions and the performance of social protection systems. High rates of under-five mortality are generally acknowledged as indicators of vulnerable healthcare systems, poor nutrition, as well as overall vulnerabilities of society. In line with the descriptive framework outlined above, it is seen that there is a tendency of greater under-five mortality to occur along with greater prevalence of mental illness, as there is a cumulative effect of early life conditions on overall mental illness. This is consistent with a life-course approach to mental illness, which views early life conditions as influencing overall mental illness. Net migration is a crucial descriptive variable in terms of capturing demographic changes and pressures on cohesion. Net migration is a function of economic opportunity, political stability, and the mechanisms and capacity of a country to integrate. The positive association between net migration and the prevalence of mental health problems could represent the psychosocial impacts of migration, as well as the challenges faced by the receiving country in the integration of migrants. At the same time, increased migration is often experienced by countries that have developed administration and health infrastructure, which could lead to increased reporting of mental health problems among both migrants and natives. The availability of safely managed drinking water services can be seen as a basic element of social infrastructure. A widespread availability of basic services is often a sign of better public health infrastructure, equity, and efficiency. On that note, the correlation between the prevalence of safe drinking water services and the prevalence of mental health problems can be constructed in a narrative manner, highlighting awareness, reporting, and coverage, as opposed to the direct impact of the former on the latter. This is especially the case in countries that have widespread coverage of basic services. Taken together, the combined significance of the social indicators highlights the importance of the Social component of the ESG framework to the issue of mental health outcomes within the broader process of social sustainability. Rather than pointing to simple or linear correlations between the indicators of social inclusion or change, the outcomes tend to suggest complex interconnections between social inclusion, institution-building, demographics, and the prevalence of mental health problems. Well-being and resilience are highlighted as key elements of social sustainability that are intricately interconnected with the labor market, early life, migration trends, and access to key services. On the whole, the results above only serve to confirm the notion that mental health must be considered from the perspective of a socially embedded phenomenon, which corresponds to the quality and inclusiveness of the social systems. Using the ESG framework, the Social dimension offers an important narrative framework for the understanding of the prevalence of mental health issues as an outcome which co-evolves with the process of social development. This offers an important narrative framework for the understanding of mental health issues from the perspective of the sustainability paradigm.

### 6.1 Hierarchical clustering for descriptive social ESG stratification and mental health patterns

The S-Social clustering analysis is a descriptive method to analyze the patterns of relationship between different aspects of social sustainability and the indicators showing the Social (S) dimension of the ESG approach. Through the use of hierarchical clustering methods, the analysis identifies different socio-economic clusters depending on the different levels of social well-being, inclusiveness, service accessibility, inequality, and institutional development in different countries. The clustering analysis is not intended to predict and optimize, but to produce a meaningful view of the relationship between social conditions and the prevalence of mental health in different social contexts. A comparative analysis of several clustering algorithms: Hierarchical, K-Means, Fuzzy C-Means, Model-Based, Density-Based, and random forest was performed to examine how the different methods might reveal the underlying structure of the data (94). This analysis suggests that the hierarchical method provides a very clear view of the global stratifications of society on the basis of generally accepted criteria of compactness, separation, and preservation of overall structure. This method is particularly effective at analyzing both homogeneous social systems and more complex ones, so that the analysis will capture the diversity of social development. The findings of the Social clustering analysis point to systematic co-occurrences between mental health prevalence and a set of wider social features, such as a level of inclusiveness, availability of basic services, level of demographic balance, and level of inequality. In this regard, it may be said that the clustering analysis highlights the relevance of the Social pillar of ESG as a framework of interpretation of collective well-being as a complex and context-dependent phenomenon, and not merely as a result of a single indicator of a social kind. In order to facilitate easier interpretation of the results, the normalized comparison table is designed by integrating measures for which higher values indicate more distinct structural separation (minimum separation, gamma correlation coefficient of Pearson, Dunn Index, Calinski & Harabasz Index) with complementary measures that reflect aspects of concentration and uncertainty (maximum diameter, entropy, Herfindahl–Hirschman Index). Due to the purely mechanical nature of the process of normalization on the 0–1 scale, more emphasis is given to separation/structure measures, with the remaining measures qualifying the results. With this descriptive approach in mind, hierarchical clustering shows high values for several separation and structure indicators, which indicates the existence of well-defined groups. K-Means also performs quite well in terms of several indicators, showing a more balanced distribution of cases in clusters, which can be useful if it is interesting to know the proportion of cases in groups. Density-Based clustering, as well as Model-Based clustering, seem less successful in identifying groups in this data. On balance, hierarchical clustering is chosen as the primary method of description because it is able to create a meaningful, organized way of describing the social stratifications relevant to the Social dimension of ESG, although it is not optimized from a technical standpoint, which is why another method, such as K-Means, is useful as a secondary point of view. Thus, method choice is based on interpretability, not technical optimality, which is why it is not optimized from a technical standpoint (95). See Table 12.

**Table 12:** Cluster validity indices across algorithms.

Indicator	Density based	Fuzzy C-Means	Hierarchical	Model based	K-Means	Random forest
Maximum diameter	1.000	0.146	0.000	1.000	0.118	0.252
Minimum separation	0.000	0.000	1.000	0.464	0.753	0.753
Pearson’s γ	0.000	0.578	1.000	0.245	0.831	0.762
Dunn index	0.000	0.050	1.000	0.289	0.748	0.706
Entropy	1.000	0.210	0.030	0.000	0.514	0.616
Calinski–Harabasz index	0.000	0.323	0.619	0.117	1.000	0.861
HH Index	0.090	0.496	1.000	0.938	0.186	0.000

This table compares six clustering algorithms based on standard validity metrics. Hierarchical and random forest methods exhibit stronger overall performance, with higher values in separation, structure (Pearson’s γ), and compactness (Dunn index). Density-based and Model-based methods show poor entropy or structure, suggesting less consistent or interpretable clustering outcomes.

The results obtained from the hierarchical clustering analysis reveal an irregular data distribution, with large variations in cluster sizes, compactness, and separation. The pattern obtained aligns with the nature of the hierarchical clustering algorithm, which generally maintains the data distribution even if it leads to irregular cluster sizes (96, 97). Descriptively speaking, the cluster sizes range from large to smaller ones. There is one large cluster, cluster 3, with a size of 87 units, accounting for almost 46% of the population, indicating the existence of a large dominant cluster with similar units along the considered variables. The other smaller clusters include clusters 6, 7, 8, and 9, each having between 5 and 9 units. The smaller ones may be considered as indicators for the existence of more specific profiles within the data (98). Clusters 1, 2, 4, and 5 lie in the middle range, thus creating a more refined segmentation between the dominant cluster and the niche profiles. The distribution of the within-cluster heterogeneity further demonstrates the descriptive nature of the clustering result. Indeed, only cluster 3 contributes around 0.539 to the total within-cluster heterogeneity, thereby confirming the prominent position of this cluster in the definition of overall variability. This is followed by the contribution of cluster 5 (0.167), and the rest of the clusters make a slightly larger contribution. It is evident that the smallest clusters make only a limited contribution to overall variability in accordance with their smaller sizes and their specific nature. This confirms the assumption that the majority of the overall variability in the hierarchical clustering solution is represented by a limited number of dominant clusters and not by an equal variability in all of them (97). Another description of the internal variability is obtained by the within-cluster sum of squares. Indeed, the largest cluster has the largest internal variability in accordance with its larger size and internal heterogeneity. On the other hand, the smaller clusters have a significantly small internal variability, thereby confirming their high internal homogeneity. This distribution confirms the assumption that the hierarchical clustering method has managed to gather highly homogeneous observations in smaller clusters while maintaining the overall and highly heterogeneous group (96). The silhouette scores provide additional information on the level of distinctiveness among the clusters. The small clusters (6, 7, 8, and 9) have relatively high values on the silhouette scores, which indicate a strong delineation from the rest of the data points. However, the large central cluster has a low silhouette score value, which implies a less distinct boundary with possible internal structures. The other clusters have medium values on the silhouette scores, which indicate acceptable but less distinct boundaries. Overall, the hierarchical clustering analysis reveals a data configuration with a large heterogeneous core cluster and several small, tightly grouped, and well-differentiated clusters. In terms of interpretation, even as the data configuration seems dominated by a central cluster with similar data points, the more distinct patterns can be found within the small clusters that identify more specific configurations on social sustainability and mental health issues (98, 99). See Table 13.

**Table 13:** Hierarchical clustering results: cluster size, heterogeneity, and cohesion metrics.

Cluster	1	2	3	4	5	6	7	8	9
Size	14	14	87	16	27	9	5	9	8
Explained proportion within-cluster heterogeneity	6.90E-02	7.10E-02	5.39E-01	1.04E-01	1.67E-01	4.00E-03	6.00E-03	2.00E-02	2.00E-02
Within sum of squares	1,36E+07	1,38E+07	1,06E+09	2,03E+07	3,27E+07	8,34E+05	1,14E+06	3,99E+06	3,90E+06
Silhouette score	4.22E-01	3.39E-01	2.21E-01	2.90E-01	2.56E-01	8.13E-01	7.34E-01	6.33E-01	5.61E-01

This table reports the main descriptive statistics of the hierarchical clustering solution. It summarizes cluster sizes, within-cluster heterogeneity, total within-cluster sum of squares, and silhouette scores. Higher silhouette values indicate stronger cluster cohesion and separation, while heterogeneity measures reflect the relative contribution of each cluster to overall variance.

Analysis of the results obtained from hierarchical clustering indicates a very unbalanced distribution of the data, with varying levels of cohesion and isolation in the clusters. This is in line with the nature of hierarchical clustering algorithms, which are known to maintain the original properties of the data even if this means having unbalanced clusters (96, 97). Descriptively speaking, the size of the clusters in the solution is quite divergent. Cluster 3 has a total of 87 observations, which is roughly 46% of the total sample and hence a large and comparable group of similar units on the chosen variables. On the other hand, the other smaller clusters of 6, 7, 8, and 9 have a considerably smaller number of units between 5 and 9. These smaller clusters may be seen as the representation of a specific configuration in the data (98). Clusters 1, 2, 4, and 5 lie in between. The composition of within-cluster heterogeneity further explains the structure of the result of the clustering. Notably, cluster 3 alone represents about 0.539 of the total within-cluster heterogeneity. This further explains the dominance of this particular cluster in influencing overall variability. Another cluster that represents a significant share of the total within-cluster heterogeneity is cluster 5 (0.167). Clusters 4, 1, and 2 represent relatively lower shares. The smallest clusters represent a limited share of overall variability in relation to their size and specificity. This further explains that overall variability in the hierarchical result is dominated by limited variability in particular clusters and not in every cluster (97). The within-cluster sums of squares are a further descriptive measure of within-cluster dispersion. The larger cluster has a large amount of dispersion, as expected, and smaller clusters are characterized by very low within-cluster dispersion, reflecting a high level of homogeneity within them. This is a clear indicator that hierarchical clustering has placed very similar observations into smaller clusters and has a more heterogeneous larger cluster as a whole (96). The silhouette scores provide additional information on the relative degree of distinction of the clusters. The smallest clusters (6, 7, 8, 9) have relatively high scores, illustrating their distinction from the rest of the dataset. The larger core cluster has a lower score, illustrating less distinctive boundaries with the possibility of sub-structures existing within the cluster. The rest of the clusters have medium scores, illustrating fair levels of distinction. Together, these hierarchical clustering outcomes characterize a configuration with a large, heterogeneous central region, as well as some smaller, more compact, and well-separated clusters. In terms of interpretability, although it is clear from this data configuration that there is a central tendency of similar data points, it is actually within these smaller clusters where the most interesting, or distinctive, configurations of social sustainability and mental health are captured (98, 99). See Table 14.

**Table 14:** Hierarchical clustering results: cluster size, heterogeneity, and cohesion metrics.

Cluster	Mental	AELC	ESRS	FERT	FOOD	GINI	EDUG	HBED	INC2	INTU	LFPR	LIFE
1	0.296	−0.012	−1.015	0.355	−0.489	0.256	0.502	−1.102	−0.185	−0.916	−0.314	−0.409
2	−1.291	0.67	−0.33	−0.241	0.687	0.008	−1.354	2.134	1.46	−1.329	0.44	−1.372
3	0.35	0.108	0.458	−0.225	0.437	−0.317	−0.431	0.12	0.393	0.653	0.376	0.035
4	−1.946	−0.862	−1.642	−0.108	−0.231	0.272	1.038	0.679	−1.225	−0.981	−1.263	−0.726
5	0.359	−1.159	0.205	−0.71	−0.676	0.471	0.679	−1.008	−0.921	−0.258	−0.52	1.25
6	0.359	1.901	0.47	3.128	0.942	0.049	1.706	−1.186	−1.609	0.38	0.466	1.222
7	0.069	2.606	−0.13	3.345	−1.172	0.709	−0.973	−0.516	1.494	−1.796	−0.604	−1.677
8	0.359	0.562	0.83	−0.701	−2.477	0.251	−0.64	−0.534	0.626	0.255	−0.697	1.125
9	−0.235	−1.09	−1.418	0.035	0.106	0.07	1.219	1.187	−0.767	0.068	0.611	−1.623

U5MR	NMIG	WATS	SANS	POP6	PDEN	OVER	UNDR	WOMP	FMRT	SCHP	SGPI	UNEM
−0.571	−0.447	0.927	−0.29	−1.012	−2.743	1.616	0.202	1.286	1.898	1.115	−2.227	−0.595
−0.359	−0.137	−0.435	−0.393	−0.65	−0.555	−0.971	−0.557	0.154	−0.432	−0.603	−0.171	−0.043
0.412	−0.165	−0.141	−0.209	0.288	0.473	0.076	0.306	−0.442	−0.178	−0.192	0.381	0.249
−0.496	−0.616	−0.444	−0.338	0.535	−0.711	−1.12	−0.759	0.865	0.405	−0.326	−0.228	−0.465
0.31	0.277	−0.226	−0.154	0.989	0.373	0.297	−0.423	−0.658	−0.432	1.518	0.585	0.559
−1.022	−0.058	−0.034	0.728	−1.678	0.661	−0.769	0.422	−0.567	−0.432	−0.743	0.776	0.029
−0.691	−0.272	−0.316	−0.485	−3.327	0.743	0.415	−0.089	4.178	2.283	−1.176	−2.438	−0.349
−1.02	−0.189	2.212	4.19	0.231	0.654	0.293	0.282	0.147	−0.432	−0.811	0.81	−1.231
−0.182	3.558	0.071	−0.566	−0.919	−1.151	−0.447	−0.495	0.643	−0.432	−0.798	−1.724	−0.978

This table reports the main descriptive statistics of the hierarchical clustering solution. It summarizes cluster sizes, within-cluster heterogeneity, total within-cluster sum of squares, and silhouette scores. Higher silhouette values indicate stronger cluster cohesion and separation, while heterogeneity measures reflect the relative contribution of each cluster to overall variance.

**Figure 4:**
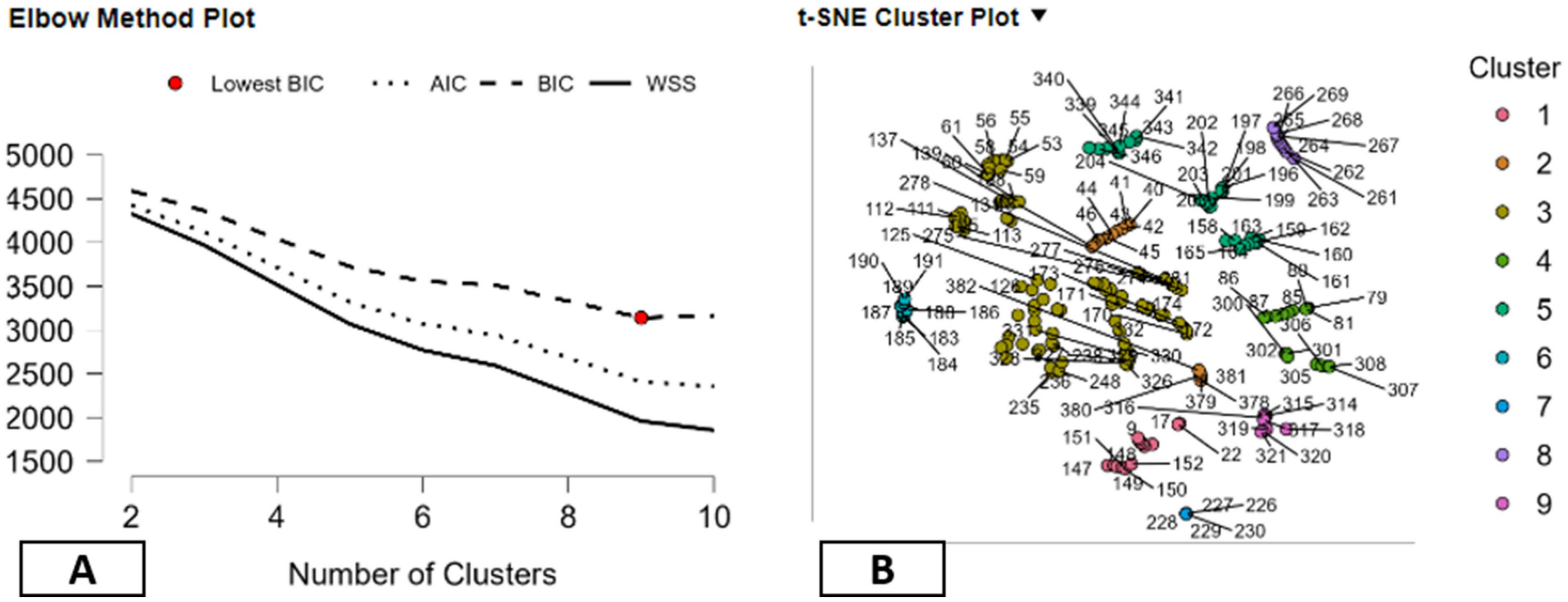
Hierarchical clustering selection and validation using elbow criteria and t-SNE visualization. **(A)** reports the elbow method with WSS, AIC, and BIC, identifying the optimal number of clusters through information criteria minimization. **(B)** presents a t-SNE projection of the hierarchical clustering solution, illustrating cluster cohesion, separation, and gradual transitions, thereby validating the selected nine-cluster structure.

The image offers an integrated representation of the selection, as well as validation, of the hierarchical solution in a manner where quantitative criteria are considered together with the graphical representation offered through cluster formation (100). In panel (A), Elbow Plot, there is a representation based on the measurement criteria of WSS, AIC, and BIC as functions of the cluster values (number of clusters). In line with the anticipated pattern, there is a gradual decrease in WSS as the values of the clusters increase, hence improving the cluster members’ homogeneity. Nonetheless, the rate at which the progress in cluster value decreases approaches a certain threshold as additional clusters are introduced—a typical scenario in large datasets of socio-economic information (101). In this regard, the point where an “elbow point” exists in these types of analyses is often not as evident, hence increasing the usefulness of information criteria, including the Bayesian Information criterion (BIC). The red point in this plot is where the lowest point of BIC exists, hence where there is a balance between cluster fit and simplicity. This further justifies the selection of the clusters based on statistical merit (100). Panel B (t-SNE Cluster Plot) offers a two-dimensional representation of the data, colored according to the nine clusters formed based on the hierarchical algorithm. Even in the absence of distance preservation, the t-SNE technique allows effective visualization of the cluster structures and cohesion, especially in the context of socio-economic and ESG analysis (102). From the plot, the separation of several clusters in the mapped, lower-dimensional space, especially those in the outer regions, emerges as compact and easily distinguished. The overlap in the central part of the plot suggests the existence of gradual transitions among the units, rather than definite boundaries among the categories, in line with the hierarchical nature of the technique, which identifies the similarity structure in the nested fashion. The simultaneous display of some large, diffused, as well as smaller, highly compact, subsets of units reflects the natural heterogeneity of the studied set. This suggests in the socio-economic and ESG studies that, despite the superficial differences, the majority of the units display generally similar properties in the context of social and mental health factors, nevertheless displaying rather sharply demarcated differences at the level of distinct subsets. The technique of hierarchical clustering allows the detection of these properties particularly effectively because of the preservation of relative similarities, as well as the absence of strict *a priori* requirements concerning the sizes and forms of the subsets (103). Taken together, these two panels offer complementary elements supporting the nine-cluster hierarchical solution. Panel A indicates there is a statistical justification for the solution based on information criteria, and Panel B presents a set of coherent and interpretable clusters when transformed into a lower-dimensional space. Taken together, this figure presents a compelling argument that hierarchical clustering is an effective tool for articulating structural information within the Social component of ESG and mental health outcomes (102, 103). See Figure 4.

Taken cumulatively, the results illustrate the clear correspondence between the indicators and the prevalence of mental health within each nation. The high explanatory power of the models and the regular significance of variables such as the labor force participation rate, the under-five mortality rate, and the availability of clean water sources illustrate that the prevalence of mental health corresponds systematically to the social conditions. Descriptively, the results illustrate that mental health trends are part of the structures of social sustainability rather than the result of health-specific processes. Simultaneously, the data also presents a pattern that requires careful analysis. For instance, while it appears that there is a positive correlation between labor force participation and the prevalence of mental illness, it might be more informative to see this as a reflection of social inclusion, the strength of social institutions, or the ability of societies to detect and report these illnesses, as opposed to a direct effect of employment on the prevalence of mental illness. The results from the clustering process further add to this complexity. The fact that a large cluster has been identified with a relatively low silhouette value suggests that a large number of observations have similar, although not identical, social profiles. This further supports the presence of sub-structures within this group, and supports the fact that the social settings associated with the prevalence of mental health issues do not have a single, homogeneous character, but rather a complex one. Several structural factors condition the interpretation of these results. Firstly, the use of country-level variables means that individual inequalities in terms of health care, education, and social services within countries are abstracted away, which can be a critical factor in individual experiences of mental health. Secondly, the lack of time-varying variables for cultural attitudes, stigma, or reporting patterns means that differences in mental health prevalence can also be a function of differences in recognition rather than individual experiences of mental health. Nevertheless, despite these limitations, the patterns identified across the social indicators all suggest that mental health is a result that develops concurrently with other processes related to social inclusion, protection, and development. Mental health seems to be connected with participation in the labor market, early childhood environments, demographics, as well as basic services provision rather than simply being a result of health factors. In this regard, the Social part of the ESG framework is a helpful narrative device for examining mental health as a phenomenon that is situated within society. A more detailed narrative can possibly be developed with the help of micro-data research on this issue.

## 7 Governance quality, institutional strength, and mental health outcomes

It is stressed in this study the importance of investigating the relationship of patterns in the quality of governance to mental health outcomes within the ESG framework, specifically in terms of institutional factors as part of social welfare. Mental health prevalence is investigated together with a group of economic and institutional factors, such as GDP growth, R&D spend, and Rule of Law, which together describe the macro-governance and development environment in which mental health outcomes are measured. Instead, these variables are used to identify the environments defined by country-level governance structures. The panel data, which includes around 30 countries over time periods of between 8 to 13 years, allows a comparison of environments defined by country-level governance structures to be made. This enables a differentiation between more stable structural elements of governance, as opposed to economic fluctuations. Overall, this helps inform an interpretive analysis for the relationship between governance, economic development, and mental health, highlighting the role that institutional quality and policy conditions play as part of the larger context for the sustainability of mental health. See Table 15.

**Table 15:** Summary of key governance, economic, and innovation indicators.

Acronym	Indicator name	Short description
Mental	Mental health prevalence	Share of the population experiencing diagnosed or self-reported mental health conditions, reflecting psychosocial well-being and the capacity of health systems to identify mental disorders.
GDPG	GDP growth	Rate of change in gross domestic product, capturing the overall expansion or contraction of economic activity in a country.
RDGD	R&D expenditure	Research and development expenditure as a share of GDP, indicating a country’s investment in innovation and technological advancement.
RULE	Rule of law	Degree to which laws are respected and enforced, reflecting institutional quality, judicial effectiveness, and protection of rights.

This table presents core indicators used to evaluate the relationships between governance quality, economic performance, innovation investment, and mental health. The inclusion of GDP growth, R&D expenditure, and rule of law offers insight into broader socio-economic and institutional conditions potentially influencing national mental health outcomes and resilience across populations.

Specifically we have estimated the following equation:


$$\text{Menta}l_{\mathrm{it}}=\alpha +\beta_{1}{\left(\text{GDPG}\right)}_{\mathrm{it}}+\beta_{2}{\left(\text{RDGD}\right)}_{\mathrm{it}}+\beta_{3}{\left(\text{RULE}\right)}_{\mathrm{it}}$$

Where i = 31 and t = 2010–2022. See Table 16.

**Table 16:** Panel regression results: effects of GDP growth, R&D expenditure, and rule of law on mental health prevalence.

Model	Fixed-effects	Random-effects (GLS) Using Nerlove’s transformation
Observations	378	
Included cross-sectional units	30	
Time-series length	Minimum 8, Maximum 13	

Dependent variable	Mental					
	Coefficient	Std. error	t-ratio	Coefficient	Std. error	z
const	2.58***	0.15	16.53	2.59***	0.54	4.78
GDPG	0.017**	0.007	2.330	0.017**	0.007	2.35
RDGD	0.32**	0.12	2.549	0.29**	0.12	2.39
RULE	0.48*	0.24	1.960	0.52**	0.22	2.36

Statistics	Test	Values	Test	Values	
	Mean dependent var	3.086746	Mean dependent var	3.086746	
	Sum squared resid	89.69390	Sum squared resid	2912.071	
	LSDV R-squared	0.970596	Log-likelihood	−922.2449	
	LSDV *F*(32, 345)	355.8792	Schwarz criterion	1868.229	
	Log-likelihood	−264.4839	rho	0.858385	
	Schwarz criterion	724.8193	S.D. dependent var	2.844515	
	rho	0.858385	S.E. of regression	2.786669	
	S.D. dependent var	2.844515	Akaike criterion	1852.490	
	S.E. of regression	0.509885	Hannan-Quinn	1858.737	
	Within R-squared	0.041287	Durbin-Watson	0.209501	
	*P*-value(F)	2.2e-243			
	Akaike criterion	594.9678			
	Hannan-Quinn	646.5038			
	Durbin-Watson	0.209501		
Tests	Joint test on named regressors Test statistic: *F*(3, 345) = 4.95254 with *p*-value = P(F(3, 345) > 4.95254) = 0.00222608		‘Between’ variance = 7.58518 ‘Within’ variance = 0.237285 mean theta = 0.950045 Joint test on named regressors - Asymptotic test statistic: Chi-square(3) = 16.3141 with *p*-value = 0.000977636		
	Test for differing group intercepts - Null hypothesis: The groups have a common intercept Test statistic: *F*(29, 345) = 304.892 with *p*-value = P(F(29, 345) > 304.892) = 9.49349e-227		Breusch-Pagan test Null hypothesis: Variance of the unit-specific error = 0 Asymptotic test statistic: Chi-square(1) = 1993.71 with *p*-value = 0		
	Distribution free Wald test for heteroskedasticity Null hypothesis: the units have a common error variance Asymptotic test statistic: Chi-square(30) = 155,088 with *p*-value = 0		Hausman test Null hypothesis: GLS estimates are consistent Asymptotic test statistic: Chi-square(3) = 6.25364 with *p*-value = 0.0999013		
	Test for normality of residual Null hypothesis: error is normally distributed Test statistic: Chi-square(2) = 551.436 with *p*-value = 1.8081e-120		Test for normality of residual Null hypothesis: error is normally distributed Test statistic: Chi-square(2) = 148.728 with *p*-value = 5.0601e-33		
	Wooldridge test for autocorrelation in panel data Null hypothesis: No first-order autocorrelation (rho = −0.5) Test statistic: F(1, 29) = 90.4831 with *p*-value = P(F(1, 29) > 90.4831) = 2.02678e-10				
	Pesaran CD test for cross-sectional dependence Null hypothesis: No cross-sectional dependence Asymptotic test statistic: *z* = 1.20749 with *p*-value = 0.227245		Pesaran CD test for cross-sectional dependence Null hypothesis: No cross-sectional dependence Asymptotic test statistic: *z* = 1.32 with *p*-value = 0.186833		

This table presents the results of fixed-effects and random-effects (GLS) panel regression models estimating the relationship between mental health prevalence and three explanatory variables: GDP growth (GDPG), R&D expenditure (RDGD), and rule of law (RULE). Both models show significant associations, with statistical tests supporting model robustness across 30 countries over 378 observations. Coefficients marked with *p* < 0.05 (), and *p* < 0.01 (*).

The above table highlights the empirical results that outline the co-variation between the Governance (G) factor of ESG and the manifestations of mental health prevalence in a panel data set of 378 observations from 30 countries, which have been under observation for a period of approximately 8 to 13 years. The results have been presented for both fixed and random effects models, and a Nerlove-type transformation has been used in both cases, which enables a comparative analysis of the results rather than a causal one. In both model specifications, the results show a high degree of consistency in terms of the signs and magnitudes of the estimates, which suggest that the co-variation between the indicators of governance and the prevalence of mental health remains similar and consistent in both model specifications. Specifically, the indicator related to the Rule of Law factor of governance consistently shows a positive co-variation with the Mental variable in both model specifications. This suggests a consistent positive correlation between high-quality institutions and good indicators of mental health, as represented by the World Health Organization’s compilation of data related to the prevalence of mental health problems among the populations of different countries, as presented by (104), and (105). Interpretively, this correlation corresponds with theoretical expectations that stronger governance structures correlate with lower levels of uncertainty, higher trust, and more predictable environments. Such factors have been widely described in the literature as contextual predictors for individual mental well-being, rather than predictors with more direct causal impacts on mental health (106). Aside from the results for the independent variables, the patterns described for the control variables appear consistent with this broader interpretational framework. GDP growth is positively related to mental health prevalence in the two equations, such that periods characterized by stronger macroeconomic performance tend to be those with more favorable mental health reporting (55, 107). Rather than implying the former, the latter may well reflect the broader social environment which accompanies periods of economic growth, including factors such as improved labor market opportunities, stable income, and reduced exposure to economic risk factors. Expenditure on research and development (R&D) is also positively related to mental health prevalence. This may reflect the former being characteristic of more innovative, institutionalized, and likely more fully developed healthcare systems, which tend to share common characteristics with stronger innovation systems (108, 109). Rather than being an independent predictor, the former may well be seen as an indicator of the broader developmental environment, rather than an independent predictor for individual mental well-being. As far as the overall fit of the models is concerned, the fixed-effects model presents an extremely high value of the LSDV R-squared, emphasizing the role of nation-specific factors in determining the prevalence of mental well-being across the globe (110). On the other hand, the low within R-squared value suggests that the changes within nations across time explain only a small fraction of the variation, which is expected due to the slow-moving nature of institutions and governance patterns at the macro-panel level of analysis (111). This further strengthens the view that the dimension of governance is structural and contextual to the notion of sustainability. Taken together, the results provide evidence for a narrative reading of the data where the Governance factor of the ESG framework represents a significant contextual institution within which the variation of mental well-being is framed. Instead of emphasizing the role of the governance factors as direct drivers of the variation of mental well-being across nations, the results illustrate the role of the quality of institutions, economic stability, and innovation factors as factors of the larger sustainability contexts within which mental well-being varies across nations. In this manner, the Governance factor of the study complements the conventional analysis of the ESG framework by broadening the framework of analysis from the realm of finance to the realm of social-mental aspects of sustainable development (104, 105, 109).

### 7.1 Governance typologies and mental well-being: a hierarchical clustering perspective

This section introduces a descriptive and interpretative analysis of the tendency for regulatory performance, governance, and economic and social contexts to cluster, and it specifically explores this tendency in terms of alignment with mental well-being patterns. Rather than testing hypotheses regarding cause-and-effect relationships, this analysis explores how different institutional arrangements empirically coalesce across nations when innovation and governance quality are taken together. To this end, a battery of indicators related to governance—Rule of Law, Government Effectiveness, Control of Corruption, and Innovation Capacity—is analyzed simultaneously. Hierarchical clustering analysis is used as an exploratory tool to classify cases by similarities in institutional design and performance. Hierarchical clustering allows for the detection of broad-based institutional regimes as well as smaller groups with similar performance, creating a stratified view of the distribution of institutional design. From a descriptive analysis, the outcome of the clustering reveals that there is a tendency toward a convergence in social trends and the quality of governance. Nations with high levels of effective institutions, regulation, and innovation are likely to be found together in clusters, which can then be described as having high levels of mental wellness and stable socioeconomic contexts. Conversely, clusters with lower levels of governance institutions are likely to depict different social and psychological vulnerabilities. The choice of hierarchical clustering is based on a comparison of various clustering algorithms, such as k-means, fuzzy c-means, density-based, model-based, and random forest algorithms, based on some structural attributes like separation, cohesion, internal consistency, and balance. In this context, hierarchical clustering is found to be more appropriate as a descriptive tool, as it maintains the natural data structure, which points to similarities among observations (96). It is worth noting that hierarchical clustering performs well on the indicators related to the separation and quality of clustering such as Minimum Separation, Pearson’s *γ* Statistic, and Dunn’s Index, which indicate a clear distinction with a degree of interpretability among the groups (99). This makes the clustering result more amenable to a narrative interpretation based on a meaningful configuration rather than a result of optimization. At the same time, other indicators point out the trade-offs in the clustering process. Even if the hierarchical clustering method has high separation and cohesion values, it has moderate results for the entropy and concentration indicators. This means that the obtained clusters have internal variability. This variability is not considered a problem but rather an expression of the complexity of the real-world governance systems, in which the institutional frameworks combine stability and variability. The comparative outcomes of the alternative approaches also support this conclusion. While k-means clustering and random forest clustering are strong on certain indicators, they are less consistent on the dimensions, with k-means clustering showing weaker separation and higher levels of entropy for random forest, indicating higher levels of dispersion within the data (100). On the other hand, hierarchical clustering provides a balanced descriptive model that is both strongly structural and flexible enough to accommodate divergent governance patterns. Taken together, the results of the clustering analysis highlight the importance of the Governance (G) dimension of the ESG framework in serving as a contextual framework through which the outcome of mental health can be described. Rather than focusing on individual indicators, the hierarchical method of analysis shows that governance quality, regulatory capacity, and innovation are interrelated in evolving in particular institutional patterns. See Table 17.

**Table 17:** Comparative performance of clustering algorithms based on internal validity metrics.

Indicator	Density Based	Fuzzy C-Means	Hierarchical	Model based	k-Means	Random Forest
Maximum diameter	0.931	1.000	0.000	0.799	0.799	0.816
Minimum separation	0.283	0.000	1.000	0.178	0.792	1.000
Pearson’s γ	0.548	0.000	1.000	0.020	0.506	0.619
Dunn index	0.268	0.000	1.000	0.178	0.632	0.791
Entropy	0.180	0.000	0.445	0.854	1.000	0.703
Calinski–Harabasz	0.000	0.734	0.706	0.653	1.000	0.654
HH index	1.000	0.673	0.322	0.074	0.000	0.182

This table compares the relative performance of alternative clustering algorithms using normalized internal validity indicators, including compactness, separation, cohesion, entropy, and concentration. Results highlight the hierarchical approach as the most structurally consistent method, achieving strong separation and cohesion while maintaining balanced cluster distributions across observations.

As illustrated by the hierarchical clustering results, the cluster structure displays a marked degree of heterogeneity, reflected in differences in cluster size, internal cohesion, and within-cluster variability (96). The distribution of observations across clusters is uneven, with a small number of large clusters coexisting alongside several much smaller groupings. In particular, clusters 1 and 3 contain the largest number of observations, with approximately 60 and 70 units respectively, whereas several other clusters include fewer than ten units. One cluster, cluster 9, is notably small, comprising only two observations. This pattern is consistent with the descriptive properties of hierarchical clustering, which is designed to preserve both broad similarities and more localized structures within the data (112). Rather than imposing uniform cluster sizes, the method allows dominant configurations to emerge alongside more specific or extreme profiles. An examination of the proportion of total heterogeneity explained by each cluster further highlights this structure. Clusters 1 and 3 account for the largest shares of overall variability, with values of approximately 0.434 and 0.382 respectively, a result that largely reflects their relative size. The remaining clusters contribute more modest shares to the overall heterogeneity, indicating that they represent narrower and more internally consistent segments of the data. This interpretation is reinforced by the within-cluster sum of squares, which is substantially higher for the larger clusters and comparatively low for the smaller ones. The low values observed for clusters 5, 6, 8, and especially cluster 9 suggest a high degree of internal homogeneity, indicating that these clusters capture very specific and tightly grouped configurations (113). Additional insight is provided by the silhouette values, which describe how distinctly each cluster is separated from the rest of the data. The smaller clusters display particularly high silhouette scores, exceeding 0.77 and reaching as high as 0.955 in the case of cluster 9, suggesting that these groupings are clearly differentiated and internally cohesive. In contrast, the larger clusters exhibit more moderate silhouette values, indicating broader and more heterogeneous configurations that overlap more with neighboring clusters. Taken together, these patterns suggest that the hierarchical clustering approach effectively reveals both dominant structural groupings and more narrowly defined profiles within the data. From a narrative perspective, the results indicate the coexistence of a few large, heterogeneous clusters that capture general institutional patterns, alongside smaller, highly cohesive clusters that reflect more specific or extreme configurations. This multi-level structure supports an interpretive reading of the results in which both common and distinctive governance and social profiles can be identified within the broader dataset. See Table 18.

**Table 18:** Hierarchical clustering structure: cluster size, heterogeneity, and cohesion.

Cluster	1	2	3	4	5	6	7	8	9
Size	60	21	70	14	7	6	7	7	2
Explained proportion within-cluster heterogeneity	0.434	0.048	0.382	0.099	0.003	0.003	0.026	0.006	5.911 × 10^−5^
Within sum of squares	252.419	27.698	222.110	57.627	1.903	1.477	15.004	3.577	0.034
Silhouette score	0.356	0.588	0.293	0.326	0.804	0.843	0.629	0.771	0.955

This table summarizes the hierarchical clustering solution by reporting cluster sizes, the proportion of within-cluster heterogeneity explained, within-cluster sum of squares, and silhouette scores. Together, these indicators assess cluster compactness, separation, and balance, highlighting both large heterogeneous groups and smaller, highly cohesive clusters within the dataset.

The Hierarchical Clustering Analysis identifies a diverse range of national profiles, each with varying combinations of mental well-being, economic conditions, governance, institutional capacity, and innovation performance (111). The fact that the cluster centers are measured in standardized deviation units from the mean allows for a comparison to be made regarding the tendency for the various factors to cluster together, rather than for any causal relationships to be inferred. Within this framework of description, Clusters 1 and 9 are identified as having profiles of comparatively lower levels of mental well-being. Both of these clusters are identified with negative values in the Mental dimension along with lower economic factors and governance capacity. In particular, Cluster 1 has factors of lower GDP growth, lower R&D spending, and lower rule of law and regulatory quality, which define the socio-institutional setting in which the factors of mental well-being are generally below the average of the sample (104). In the case of Cluster 9, it has factors of lower corruption control, regulatory quality, and institutional capacity. At the other end of the distribution, Cluster 2 corresponds to a profile where the indicators of mental well-being are above the mean of the sample and combine with good economic performance, high investment in R&D, and good governance indicators on various dimensions such as rule of law, quality of regulation, and government effectiveness. This cluster is more indicative of a pattern where good economic, institutional, and innovation factors co-exist with high self-reported levels of mental well-being (108). The profile emerging from Cluster 3 is more moderate and well-balanced. The indicators for mental well-being tend to be slightly above the average, while the economic, governance, and innovation factors tend to be around the mean. This cluster corresponds to an institutional and economic environment that is stable, where the mental well-being outcomes tend to be neither outstandingly high nor low, but rather correspond to an intermediate environment. Other clusters tend to reveal more complex or subtle patterns. Thus, for instance, in the case of Cluster 4, there are moderate positive indicators of mental well-being together with weaker government effectiveness and less innovation, which can be taken to mean that favorable outcomes in some areas can coexist with difficulties in other areas. Likewise, in the case of Cluster 5, there are high R&D expenditure and indicators of mental well-being, but persistent weaknesses in regulatory quality. Clusters 6 and 7 are positioned in an intermediate manner within this distribution as a whole. The mental well-being variables in these two clusters are lower than the sample mean, although this is less true than in the more disadvantaged mental well-being profiles. The mental well-being profiles in clusters 6 and 7 are a result of unique combinations of advantages and disadvantages, such as a relatively stable political system and less stable economy/governance, among others. Taken cumulatively, the outcomes of hierarchical clustering help in forming a descriptive summary of the typical alignment of mental well-being outcomes with different combinations of quality of governance, performance of institutions, economic conditions, and innovation capabilities of countries. Instead of signaling a singular developmental path, the outcomes point toward the existence of different structural formations in which the outcomes of mental well-being are situated. In the larger framework of ESG, the outcomes point toward the significance of the role of innovation and governance in acting as recurring features in the distribution of mental well-being outcomes across countries, and at the same time, the diversity of different pathways through which the outcomes are experienced (104, 108, 111). See Table 19.

**Table 19:** Cluster characteristics by mental health and governance indicators.

Clusters	Mental	CORR	GDPG	GOVE	PATR	POLS	REGQ	RDGD	RULE	SCIA	LGRI	VOIC
Cluster 1	−1.001	0.222	−1.119	−0.216	−0.404	−0.290	−0.779	−0.789	−1.134	−1.092	−0.569	−1.161
Cluster 2	1.942	−0.522	1.690	0.091	−0.388	−0.140	1.016	1.647	1.280	1.666	0.093	1.238
Cluster 3	0.261	0.180	0.304	0.632	0.529	−0.242	0.559	−0.122	0.524	0.357	−0.223	0.463
Cluster 4	0.164	0.371	0.263	−1.669	−1.004	−0.335	0.568	−0.246	0.312	0.400	−0.332	0.657
Cluster 5	0.782	0.301	1.111	−0.062	−1.014	−0.123	−1.499	3.322	0.877	0.821	0.006	0.428
Cluster 6	−0.018	−0.946	0.108	−1.669	−0.651	1.387	0.370	0.269	0.277	0.048	3.109	0.710
Cluster 7	−1.132	−0.692	−0.790	0.340	−0.153	4.825	−1.245	−0.029	−1.193	−1.256	2.593	−1.395
Cluster 8	0.547	−0.339	0.859	−0.464	3.010	0.088	0.179	0.133	0.477	0.761	2.305	0.759
Cluster 9	−1.265	−4.699	−1.109	0.741	0.187	0.089	−2.950	−0.436	−1.343	−1.297	−0.253	−0.406

**Figure 5:**
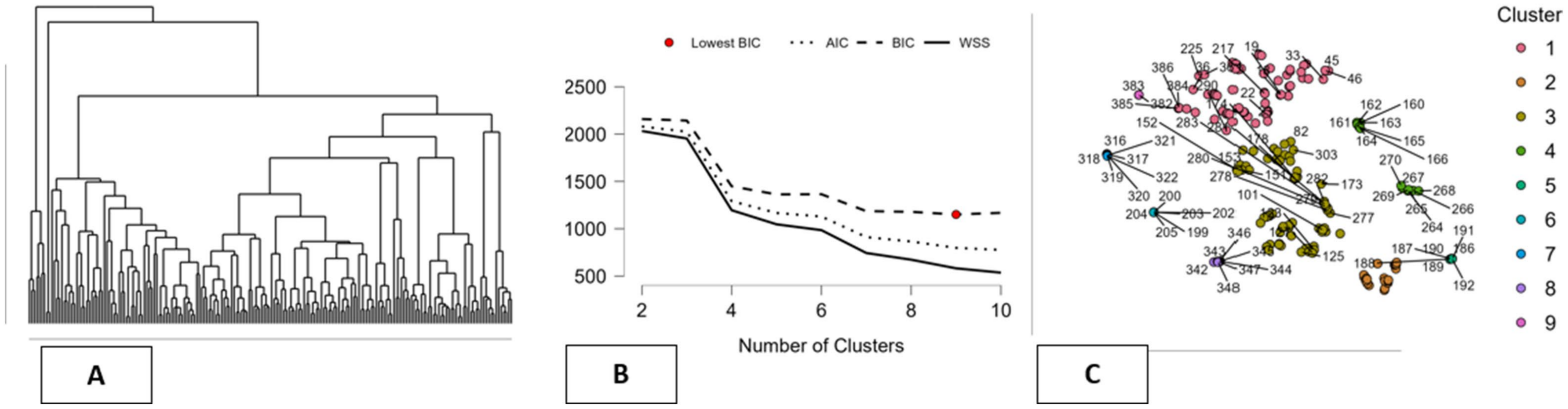
Hierarchical clustering solution: structure, selection, and validation. This figure integrates three panels to assess the hierarchical clustering outcome. The dendrogram **(A)** reveals complex structure; the elbow plot **(B)** identifies nine as the optimal cluster number using BIC; and the *t*-SNE plot **(C)** visually confirms distinct clusters. Together, they validate the clustering solution for socioeconomic ESG analysis.

The following figure presents a combined descriptive analysis of the hierarchical clustering solution by incorporating information available through the dendrogram, criteria used for identifying the number of clusters, as well as the final representation of the cluster structure in a reduced dimensional space (103). Taken together, these three sub-figures offer complementary insights into how heterogeneity can be structured within the data, as opposed to being used as a means of formal validation. Figure A presents the hierarchical dendrogram, where the progressive merging of observations as a function of dissimilarity level can be observed. That several elongated branches exist before the final merging points toward a data structure where multiple observation-level clustering takes place, pointing toward a multi-level data structure where a combination of more defined groups as well as larger groups exist, as opposed to a data structure where a few homogeneous groups exist (100). Descriptively, it can be seen how the dendrogram points toward an unbalanced distribution of similarities between observations, where some groups exist at a higher level of similarity while some groups exist at higher dissimilarity levels. Figure B presents a complementary analysis where the evolution of criteria used for clustering as a function of the number of clusters can be seen. With a progressive increase in the number of clusters, the Within-Cluster Sum of Squares (WSS) continues to decrease at a diminishing rate, while information criteria (AIC&BIC) continue to show a flattening trend where the minimum BIC value continues at a relatively large number of clusters, as indicated by the red spot on the graph. Rather than pointing toward a clear-cut threshold, this trend continues to suggest how structural detail continues to be discovered as a function of increased granularity, supporting a descriptive analysis where a heterogeneous data structure continues to exist (114). Figure C presents a two-dimensional representation of the hierarchical clustering solution where a more intuitive representation of how observations exist at different clusters can be observed. At this reduced space, several clusters continue to exist as more defined groups where some groups continue to show overlapping or elongated structures, as per the nature of hierarchical clustering where no specific assumption of equal-sized or spherical-shaped clusters exist (114). Rather, the representation depicts different levels of internal cohesion and separation among the clusters in accordance with the uneven structure that can be seen in the dendrogram. In sum, the three representations describe a set of data that contains multi-level heterogeneity. In particular, the dendrogram depicts nested structures of similarity, while the conditions for clustering describe the level of explanatory detail that cumulates with increasing numbers of clusters, and finally, the low-dimensional representation depicts these structures in visual form. In terms of narrative explanation, the graph shows that the hierarchical solution of the clustering problem decomposes the data into chunks that correspond to broad patterns and specific ones in particular (100, 103). See Figure 5.

The study highlights that there is consistent and strong evidence that supports a positive correlation between institutional quality, as defined by Rule of Law, and mental health outcomes. Furthermore, both fixed and random effects models support the implication that governance is a fundamental mediator of well-being. However, there are some specific limitations of interpretation and theoretical implications that should be considered. First of all, within-R-squared statistics are relatively low, suggesting that most of the valuable variations are due to cross-sectional variations and not improvements over time. This implies that countries that perform better in terms of institutional quality perform well in terms of outcomes, although improvements over time might lack effect. Moreover, there is some risk of endogeneity, as better outcomes might improve institutions through enhanced stability and trust. Third, although the Rule of Law variable is theoretically profound, there is a risk of oversimplification of institutions. Clustering analysis introduces more specific variables about diverse institutional patterns and corresponding distinct variations of mental health. However, there is some risk of having very divergent and large clusters (such as Cluster 3): and there is some risk of substantial gaps between Cluster 1 and Cluster 2. This implies that there could be further, non-linear dynamics at play. Overall, there is some evidence that governance is fundamental to variations of mental health outcomes. However, there might be some mediation of institutional quality through some additional factors such as innovation and development.

## 8 Mental health as a structural outcome of environmental, social, and governance dynamics

The current research provides a comprehensive descriptive analysis of how outcomes related to mental health issues fit into the environmental, social, and governance factors of sustainability, using a systematic literature review as well as a cross-national analysis of 31 countries between 2010–2022 (115). Contrary to how mental health issues might be viewed as a secondary or medical topic, the analysis places the prevalence of mental health issues into a structural framework defined by factors of the environment, social structures, and settings (116). One key descriptive characteristic that has been identified is the evident heterogeneity in the prevalence of mental health issues across different countries as well as across time. This heterogeneity does not appear to be random; rather, it is indicative of cross-national differences. Nations that have been experiencing radical changes in the economic, social, or environmental sphere tend to have higher levels or an increasing trend with respect to the prevalence of mental health issues, while those with more stable development patterns tend to have less variability (117). Concerning the Environmental component, the results indicate a systematic relationship between mental health prevalence rates and energy use patterns as well as environmental management practices. A greater reliance on coal-driven electricity sources and overall per capita energy consumption are generally associated with a greater prevalence of mental health issues, as seen in environments characterized by environmental strains, pollution, and resource-driven development patterns (118). At the same time, a greater degree of energy efficiency is generally associated with more desirable mental health patterns, as seen in environments characterized by a more managed use of resources (119). The relationship between mental health prevalence rates and other factors such as the use of renewable energy sources and environmental protected areas is more complex from a descriptive standpoint. These factors are seen as indicative neither of environmental degradation nor resource strains but rather as indicative of transition processes within the realm of sustainability, improved monitoring capacities, as well as greater awareness regarding mental health conditions (115). The Social factor further situates mental health outcomes in the context of life-course processes. The synthesis underscores the ways in which insecurity in the economy, housing and food insecurity, insecurity in the context of migration, and social exclusion are generally co-occurring with elevated levels of mental distress (116). On the other hand, societies which have stronger social infrastructure and welfare structures and which have high levels of community engagement generally have patterns which correspond to stronger mental health and stronger mental health profiles (117). These findings underscore the narrative approach to mental health as a social embedded outcome. Governance is revealed as a cross-cutting contextual factor that conditions the relationship between environmental and social factors and mental health outcomes. Descriptive trends appear to suggest that variations in the quality of institutions shape the impacts of sustainability transitions, social policies, and economic shifts on populations. In the absence of strong regulatory and transparent environments, and in the absence of inclusivity in decision-making, environmental and social stresses are likely to co-occur with vulnerability in mental health outcomes. In contrast, environments that are more inclusive and transparent in their governance are likely to see trends of low uncertainty and strong institutional support, reflected in favorable mental health outcomes (119). Cumulatively, the results point to a single descriptive truth: the prevalence of mental health is deeply entwined with the environmental, social, and governance aspects of sustainability. By incorporating mental health into the realm of ESG-defined sustainability frameworks, mental health can now be considered both the product of structural factors and the informative indicator of larger societal well-being. From this vantage point, mental health offers a rich narrative framework through which the human significance of the sustainability trajectory may now be better appreciated (115, 116, 119). See Table 20.

**Table 20:** Integrated summary of ESG dimensions and mental health evidence.

ESG Component	Descriptive patterns in mental health outcomes	Evidence from panel data analysis	Evidence from clustering analysis
Environmental (E)	The environmental dimension is characterized by recurring patterns linking mental health prevalence with energy use, production structures, and environmental management. Contexts marked by higher energy intensity and environmentally unsustainable production tend to co-occur with higher reported levels of mental health conditions, while settings with greater energy efficiency display more favorable profiles. Periods of rapid environmental transition are frequently associated with heightened psychological sensitivity.	Panel estimates reveal stable and statistically significant associations between environmental and energy indicators and mental health prevalence across countries and over time. These associations vary considerably across national contexts, highlighting strong structural heterogeneity rather than uniform trends.	Environmental clustering identifies groups of countries sharing similar sustainability and energy profiles. Clusters representing advanced stages of environmental transition often show higher observed mental health prevalence, which is interpreted as reflecting greater institutional capacity for monitoring, diagnosis, and reporting rather than underlying deterioration in mental well-being.
Social (S)	Social conditions emerge as a central contextual layer shaping mental health patterns. Higher prevalence of mental health conditions tends to co-occur with settings characterized by economic insecurity, social inequality, and uneven access to basic services, whereas more inclusive social systems and welfare arrangements are associated with lower psychological vulnerability.	Panel data analysis shows that indicators related to social inclusion, welfare provision, and living conditions account for a substantial share of cross-country variation in mental health prevalence. These associations are predominantly long-term and cumulative, reflecting persistent social structures.	Social clustering distinguishes countries with relatively strong social cohesion, access to services, and institutional support from those marked by fragmentation and exclusion. These distinct social profiles align with differing levels and patterns of mental health prevalence across clusters.
Governance (G)	Governance quality appears as a cross-cutting contextual factor shaping how environmental and social conditions are reflected in mental health outcomes. Countries characterized by stronger institutional capacity, regulatory effectiveness, and inclusive governance tend to display more stable mental health profiles, particularly during periods of social and environmental change.	Panel models indicate robust associations between governance indicators and mental health prevalence. Governance variables systematically align with cross-country differences, suggesting that institutional settings condition how environmental and social pressures are experienced and recorded.	Governance-based clustering clearly separates countries with stronger institutional quality and adaptive capacity from those with weaker governance structures. These clusters correspond to distinct configurations of systemic psychological stress and resilience.

This table provides a narrative synthesis of the relationships between environmental, social, and governance (ESG) dimensions and mental health outcomes. Evidence from panel econometric analysis and clustering techniques is triangulated to highlight recurring cross-country patterns, structural heterogeneity, and contextual configurations, rather than causal effects. The table emphasizes how mental health prevalence is embedded within broader sustainability profiles and institutional settings.

## 9 Policy implications within a descriptive ESG–mental health framework

As stated in this section, the policy implications are deliberately informed by the descriptive and exploratory nature of the empirical approach used in the current study. In other words, since the empirical analysis in the current study uses correlation panel regressions and clustering analysis, and does not conduct causal identification approaches like instrumental variables, policy shocks, and quasi-experimental methods, the results obtained cannot be used to inform causality and prescriptive policy assessment, as stated by Kim and Yang in 2025. In other words, the implications presented in this section should be considered policy-relevant findings, which are informed by descriptive associations, and should not be considered as empirical evidence regarding the effectiveness of interventions. In empirical terms, the analysis in the study finds regular co-occurrences and heterogeneities in the relationship between mental health prevalence and environmental, social, and governance (ESG) metrics in different countries, as stated in (17). In other words, there is a regular and systematic correlation in the analysis, indicating that in countries with high prevalence levels of mental health, there are also high levels of sustainable transitions, strong social infrastructure, and high capacities in governance, as stated in Zhang et al. in 2025. However, it is important to note that such associations should not be construed as implying negative effects of institutional development progress or sustainability progress on mental health. On the contrary, such associations can be more validly interpreted in terms of differences in the capacity for diagnosis, awareness of institutions, reporting, as well as the social perception of mental health as a public matter. From the analysis perspective, the main policy implications of the findings are in terms of the focus on the need for coordination and integration, as opposed to the focus on the design of interventions. From the findings, the main policy implications of the study results are that the outcomes of mental health are likely to be found at the nexus of environmental transition, social protection, as well as the quality of governance, in terms of the challenges of viewing mental health as a sectoral policy focus (120). From the analysis perspective, the prevalence of mental health can be validly interpreted as a useful contextual indicator of the institutional as well as the social circumstances that are attendant upon the process of sustainability transitions (15). The evidence here is therefore in support of the inclusion of considerations of mental health within sustainability-influenced policy frameworks in relation to areas of transition in the environment and society, without necessarily assuming direct causal relationships (2). In this particular aspect, therefore, the outcome of mental health can be considered as a kind of “lens” that policymakers can use to foresee possible psychosocial stresses that are bound to come with structural transformations and institutional reforms in society without necessarily being used to judge the effectiveness of particular policy interventions (17, 120). In conclusion, therefore, the policy implications of the current research are therefore intended to be contextual and non-prescriptive in line with the nature of the empirical approach adopted.

## 10 Limitations in scope, measurement, and analytical framework

Nonetheless, some limitations must be taken into account in the process of result interpretation. Firstly, the data employed in this analysis is based on secondary sources. Although the employed sources are reliable and common in global studies, the difference in the process of data collection, reporting, and awareness levels across different countries must be taken into account (121). Indicators for the prevalence of mental health issues, despite being standardized, could differ considerably based on the differences in the perception of mental health issues, the degree of stigma, and the ability to identify mental health issues (121). This could mean that the increased prevalence rates in some countries could be an indication of more sophisticated systems in place, as opposed to the actual mental health status. The second limitation relates to the level of aggregation used in the empirical test. Country-level variables are suitable for cross-country ESG analysis, but they always obscure considerable in-country heterogeneity (122). Outcomes of mental health are determined by a broad spectrum of interacting variables, such as income, gender, age, ethnic status, and geographic location, that cannot be distinguished separately in the analysis of macro-level variables. Likewise, the ESG variables obscure the in-country inequalities in terms of vulnerability, social service delivery, and institutional performance. Therefore, the results should be interpreted exclusively in the macro-level context and should not be projected to the individual-level or the community-level process (123). The next set of limitations is related to ESG itself. While ESG indicators form a widely accepted framework of structuring integration of environmental, social, and governance aspects, ESG itself is still a dynamic concept, to some extent disputed (124, 125). “Crucial aspects of sustainability, such as informal governance, social capital, or social cohesion, or more generally, subjective experiences of the natural environment, remain hard to grasp using available quantitative indicators.” In methodology, panel data analysis uncovers statistical correlations but does not imply causations. While fixed- or random-effects models mitigate concerns about time-constant country-specific factors, concerns about endogeneity remain difficult to eliminate entirely (122). “Reverse causality may arise, as mental illness may impact economic performance or stability, which, in turn, may impact ESG performance. Additionally, unobservable variables such as culture, stigma, or unobservable policy interventions may impact both mental illness outcomes and ESG performance simultaneously” (121). However, there are further constraints that come with the analysis of the clusters. Although various methods of cluster analysis and validation indices have been used in this research, it should be noted that cluster analysis tends to oversimplify complex realities (124). Within each country’s cluster, there can be great differences on particular dimensions of sustainability transitions and mental health, and it is important to remember that the boundaries of these clusters are merely indicative. In relation to the time dimension of the research’s database, it should be noted that while it spans more than a decade, sustainability transitions and the dynamics of mental health can be processes that span much longer periods of time. The effects of institutional reforms, climate conditions, or interventions in social policy on mental health can take longer to manifest themselves in reality than the time span of the database (122). Finally, it should be noted that while this research has adopted a narrative literature review that has permitted it to synthesize in a broad and integrative fashion, it has also done so in a selective fashion. In other words, by focusing on particular databases, keywords, and time spans, it is possible that important contributions have been excluded that come from other disciplines or other periods of the literature (124, 125). Future research would be improved by expanding on the time horizon of the research database and including other research databases in the synthesis. Also important would be the complementary use of qualitative research methods (123). Taken together, these shortcomings do not diminish the relevance of the research. Rather, they illuminate the complexity of integrating mental health considerations within the ESG framework and suggest the need for further research that utilizes micro-data, a long-time horizon, as well as a mixed-methods approach to further elucidate mental health within sustainable development processes (121, 122).

## 11 Conclusion

This paper contributes to the burgeoning body of research which conceives of mental health as a fundamental component of sustainability, as opposed to a residual or sectoral outcome. In particular, by locating mental health research within the paradigm of environmental, social, and governance performance, this research seeks to rectify what is currently a problem of fragmentation within the current body of research, whereby psychological well-being is often conceived of as a secondary outcome of either Environmental, or Social, or Institutional factors. One of the major contributions of this study is the integrated research design. This study combines a narrative review of the dimensions of ESG with a cross-national empirical study. The narrative review allows for the organized integration of the discussions of mental health within the various dimensions of sustainability without necessarily applying a common conceptual or causal framework. Through the assessment of the three dimensions of ESG individually, the narrative review presents how mental health factors arise individually within the various dimensions of sustainability. This narrative synthesis can be supplemented by the empirical part in terms of examining cross-country patterns based on panel data for 31 countries between 2010–2022. Instead of examining individual countries or specific points in time, this study reveals structural differences between mental health prevalence and some ESG-related indicators. This focuses more on differences within national contexts without attempting to establish causal links. Another original aspect is the use of clustering analysis in combination with panel regressions. By using unsupervised learning in the form of clustering methods, the research is able to categorize the countries based on their common ESG and mental health factors. This results in the creation of clusters which show how different countries have different paths for the relationship between the conditions of sustainability and mental health outcomes based on their capacity and social protection structures. From a conceptual perspective, the issue of mental health is considered in the study as both an outcome and a signal of ESG performance. Differences in the prevalence rates of mental health are considered to signal differences in environmental, social, and governance factors, as opposed to health outcomes per se. In this case, the issue of governance quality is considered to be a crucial contextual issue in shaping the relationship between environmental and social factors and their outcomes in terms of mental health. In conclusion, the research gives a holistic, descriptive explanation of the link between mental health and ESG factors. Via narrative synthesis, panel analysis, and clustering, the research illustrates that mental health needs to be viewed as a fundamental aspect of sustainability discourse. By placing mental wellness at the focal point of the ESG model, the study helps create a more human-centric view of sustainability that recognizes the social-institutional context surrounding psychological outcomes.

## Glossary

ACFT: Access to clean fuels and technologies for cooking
AELC: Access to electricity
ASNR: Adjusted savings: natural resources depletion
ASFD: Adjusted savings: net forest depletion
AGLD: Agricultural land
AGVA: Agriculture, forestry, fishing value added
FWWD: Annual freshwater withdrawals
CO2P: CO₂ emissions per capita
CORR: Control of Corruption
CDD: Cooling Degree Days
ESRS: Economic and Social Rights Score
ELCL: Electricity from coal
ENIM: Energy imports
ENIN: Energy intensity
ENPC: Energy use per capita
FERT: Fertility rate
FOOD: Food production index
FRST: Forest area
FOSS: Fossil fuel consumption
GDPG: GDP growth
GINI: Gini index
GOVE: Government Effectiveness
EDUG: Government expenditure on education
HI35: Heat Index 35
HDD: Heating Degree Days
HBED: Hospital beds
INC2: Income share lowest 20%
INTU: Internet users
LFPR: Labor force participation rate
LST: Land surface temperature
WSTR: Water stress level
LIFE: Life expectancy
CH4P: Methane emissions per capita
U5MR: Under-5 mortality
NMIG: Net migration
N2OP: Nitrous oxide emissions per capita
PATR: Patent applications (residents)
WATS: Safe drinking water
SANS: Safe sanitation
PM25: PM2.5 exposure
POLS: Political stability
POP6: Population 65+
PDEN: Population density
POVR: Poverty headcount
OVER: Overweight prevalence
UNDR: Undernourishment prevalence
WOMP: Women in parliament
FMRT: Female/male LFPR ratio
REGQ: Regulatory quality
RELE: Renewable electricity
RENC: Renewable energy consumption
RDGD: R&D expenditure
RULE: Rule of law
SCHP: Primary school enrollment
SGPI: School GPI
SCIA: Scientific articles
SPEI: SPEI drought index
LGRI: Legal rights strength
PROT: Protected areas
TREE: Tree cover loss
UNEM: Unemployment rate
VOIC: Voice and accountability
